# Impact of lymph node metastasis on immune microenvironment and prognosis in colorectal cancer liver metastasis: insights from multiomics profiling

**DOI:** 10.1038/s41416-024-02921-2

**Published:** 2025-01-03

**Authors:** Yueyang Zhang, Deng Wu, Zhen Zhang, Jian Ma, Shuai Jiao, Xiaolong Ma, Jiangtao Li, Yongsheng Meng, Zhixun Zhao, Haipeng Chen, Zheng Jiang, Guiyu Wang, Haiyi Liu, Yanfeng Xi, Haitao Zhou, Xishan Wang, Xu Guan

**Affiliations:** 1 Department of Colorectal Surgery, National Cancer Center/National Clinical Research Center for Cancer/Cancer Hospital, Chinese Academy of Medical Sciences and Peking Union Medical College, Beijing, China; 2 College of Biomedical Information and Engineering, Hainan Medical University, Haikou, China; 3 Department of Pathology, Shanxi Province Cancer Hospital/Shanxi Hospital Affiliated to Cancer Hospital, Chinese Academy of Medical Sciences/Cancer Hospital Affiliated to Shanxi Medical University, Taiyuan, China; 4 Department of Colorectal Surgery, Shanxi Province Cancer Hospital/Hospital Affiliated to Cancer Hospital, Chinese Academy of Medical Sciences/Cancer Hospital Affiliated to Shanxi Medical University, Taiyuan, China; 5 Department of Colorectal Surgery, Nanjing Drum Tower Hospital, the Affiliated Hospital of Nanjing University Medical School, Nanjing, China; 6 Department of Pathology, National Cancer Center/National Clinical Research Center for Cancer/Cancer Hospital, Chinese Academy of Medical Sciences and Peking Union Medical College, Beijing, China; 7 Department of Tumor Biobank, Shanxi Province Cancer Hospital/ Shanxi Hospital Affiliated to Cancer Hospital, Chinese Academy of Medical Sciences/ Cancer Hospital Affiliated to Shanxi Medical University, Taiyuan, China; 8 Department of Colorectal Cancer Surgery, the Second Affiliated Hospital of Harbin Medical University, 246 Xuefu Road, Nangang District Harbin, China

**Keywords:** Metastasis, Colorectal cancer

## Abstract

**Background:**

This study aimed to investigate the prognostic impact of lymph node metastasis (LNM) on patients with colorectal cancer liver metastasis (CRLM) and elucidate the underlying immune mechanisms using multiomics profiling.

**Methods:**

We enrolled patients with CRLM from the US Surveillance, Epidemiology, and End Results (SEER) cohort and a multicenter Chinese cohort, integrating bulk RNA sequencing, single-cell RNA sequencing and proteomics data. The cancer-specific survival (CSS) and immune profiles of the tumor-draining lymph nodes (TDLNs), primary tumors and liver metastasis were compared between patients with and without LNM. Pathological evaluations were used to assess immune cell infiltration and histological features.

**Results:**

The CRLM patients with LNM had significantly shorter CSS than patients without LNM in two large cohorts. Our results showed that nonmetastatic TDLNs exhibited a greater abundance of immune cells, including CD4+ T cells, CD8+ T cells, and CD19+ B cells, whereas metastatic TDLNs were enriched with fibroblasts, endothelial cells, and macrophages. Immunohistochemical analysis confirmed elevated levels of CD3+ T cells, CD8+ T cells, and CD19+ B cells in nonmetastatic TDLNs. The presence of nonmetastatic TDLNs was associated with enhanced antitumor immune responses in primary tumors, characterized by a higher Klintrup–Makinen (KM) grade and the presence of tertiary lymphoid structures. Furthermore, liver metastasis in patients with nonmetastatic TDLNs were predominantly of the desmoplastic growth pattern (dHGP), while those with metastatic TDLNs were predominantly of the replacement growth pattern (rHGP).

**Conclusions:**

This research highlights the adverse prognostic impact of LNM on patients with CRLM and reveals potential related mechanisms through multiomics analysis. Our research paves the way for further refinement of the AJCC TNM staging system for CRLM in clinical practice.

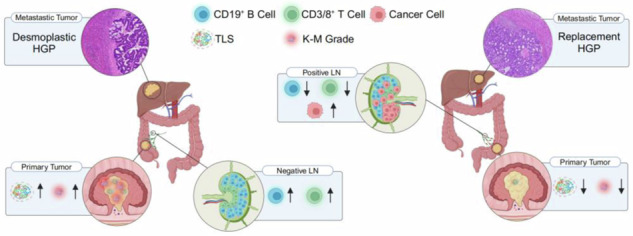

## Introduction

Colorectal cancer (CRC) is the second leading cause of cancer-related mortality globally [1]. The liver is the most common organ of distant metastasis in CRC [2]. However, the metastasis status of tumor-draining lymph nodes (TDLNs) in patients with colorectal cancer liver metastasis (CRLM) has not received adequate attention. Previous studies have indicated that 27.1–38.2% of CRLM patients do not exhibit lymph node metastasis (LNM) [3, 4]. The significance of LNM is often overlooked in prognostic evaluations for CRLM patients based on the TNM staging system [5–7], and the presence or absence of LNM does not affect the decision-making of treatment plans. However, several studies have shown that despite the high mortality rate of patients with CRLM, patients with CRLM without LNM tend to have a better prognosis than those with LNM [3, 4].

TDLNs are the primary sites for the LNM of tumor cells. The TLDNs play a pivotal role in the intricate interplay between tumors and the host immune system, exerting profound immunological effects on tumor progression [8–12]. Recent studies have highlighted the concept of the “tumor–immunity cycle,” wherein continuous crosstalk between tumor cells and immune cells within TDLNs regulates the immune response against cancer [13]. Within the tumor microenvironment, TDLNs act as critical sites for the priming and expansion of immune cells, particularly T cells [14, 15]. Moreover, TDLNs facilitate the recruitment and activation of various immune cell subsets, including dendritic cells, B cells, and natural killer cells, which collectively contribute to the antitumor immune response. This dynamic interaction between tumor cells and immune cells within TDLNs shapes the overall immune landscape of the tumor microenvironment, influencing disease progression and patient outcomes. Disruption of the cell cycle, often observed in TDLN metastasis, can compromise antitumor immunity, facilitating tumor evasion and progression [16]. Understanding the intricate immunological dynamics within TDLNs is paramount for deciphering the mechanisms underlying CRC metastasis and devising novel immunotherapeutic strategies.

Since TDLNs play key roles in the immune response, influencing the efficacy of treatments for CRLM, and LNM is often overlooked in CRLM prognosis evaluation, these compelling findings prompted us to assess the role of TDLNs in CRLM. Leveraging advanced techniques such as single-cell analysis and multiomics approaches can provide deeper insights into the complex cellular and molecular interactions occurring within TDLNs. To the best of our knowledge, no study has explored the mechanisms by which LNM affects the prognosis of CRLM. These findings could be very valuable for refining the TNM staging system for CRC and paving the way for more effective immunotherapeutic interventions for CRC management, which is the underlying goal of our research.

In this study, we leveraged data from two large population-based cohorts and integrated multiomics resources, including bulk and single-cell transcriptomics, proteomics, and pathomics data, to elucidate the intricate mechanisms underlying the impact of LNM in CRLM patients. Our findings will improve our understanding of CRC progression and may guide refinement of the TNM staging system. This study also provides valuable clinical and mechanistic insights obtained via multiomics profiling of TDLNs and primary and metastatic tumors that can guide clinical management and prognosis assessment.

## Methods

### Ethics statement

The clinicopathological data collection for this study was conducted according to the principles of the Declaration of Helsinki. All tissue samples, including paired primary tumor, liver metastasis, and TDLN samples, were obtained in accordance with national guidelines. These tissues were collected from surgical specimens after macroscopic examination by two pathologists. For each specimen, a fragment was utilized for transcriptomic and proteomic sequencing. The remainder of the tissue was immediately used to generate formalin-fixed and paraffin-embedded (FFPE) samples for histopathology. All patients provided signed informed consent, and the study was evaluated and approved by the institutional review board (IRB) of the National Cancer Center/Cancer Hospital, Chinese Academy of Medical Sciences & Peking Union Medical College and Shanxi Hospital Affiliated to Cancer Hospital, Chinese Academy of Medical Sciences & Peking Union Medical College.

### Patient cohorts

**Fig. 1:**
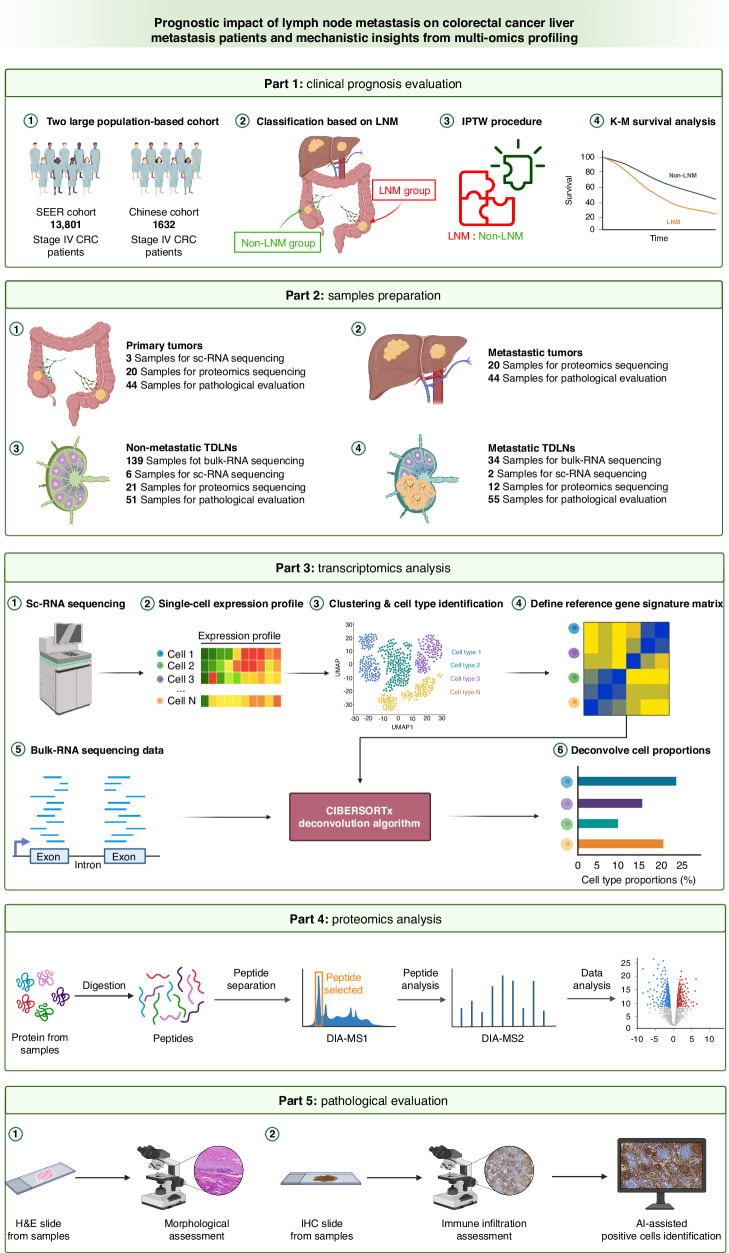
Study workflow. SEER Surveillance, Epidemiology, and End Results, LNM lymph node metastasis, IPTW inverse probability of treatment weighting, K‒M Kaplan–Meier, sc-RNA single-cell RNA, DIA data-independent acquisition, MS mass spectrometry, H&E hematoxylin & eosin staining, IHC immunohistochemistry, AI artificial intelligence.

The workflow of this research is presented in Fig. 1. The US Surveillance, Epidemiology, and End Results (SEER) cohort consisted of patients with CRLM who underwent surgical resection between January 2010 and December 2017. Patient samples were uniformly reviewed and staged according to the 8th edition of the AJCC TNM staging system. A multicenter Chinese cohort comprising CRLM patients from four Chinese tertiary centers was enrolled based on the same criteria. To ensure the accuracy of the TDLN status of the enrolled patients, the number of TDLNs examined in all patients was greater than 12. The baseline characteristics of these two cohorts are summarized in Supplementary Table 1. Furthermore, the baseline characteristics of the two groups were matched by Inverse Probability of Treatment Weighting (IPTW) to reduce selection bias [17].

### Survival analysis

The primary outcome was cancer-specific survival (CSS), which was defined as the time interval from diagnosis until cancer-specific death or the end of follow-up in the Chinese cohort, and CSS was defined using SEER cause-of-death codes in the SEER cohort [18]. Multivariate analysis was performed using the Cox proportional hazards regression model to adjust for confounding factors and further elucidate the impact of LNM on survival. Survival analyses were performed using the Kaplan‒Meier method, and differences between groups were assessed with the log-rank test.

### Multiomics sequencing sample preparation

Preoperative adjuvant therapies such as radiotherapy and chemotherapy can affect the status of the TDLN. Consequently, obtaining samples of TDLNs with clear metastasis status can be difficult since adjuvant treatment can affect metastasis status. To compare the transcriptomic differences between metastatic and nonmetastatic TDLNs, we collected 173 TDLNs from 82 patients with stage I-III CRC at the National Cancer Center for comprehensive bulk RNA sequencing analysis. Among these TDLNs, 139 were nonmetastatic, and 34 were metastatic.

To further clarify the impact of tumor metastasis on the cellular components of TDLNs, we collected 3 primary tumor tissues and 8 paired TDLNs from 3 CRC patients, one with LNM and two without LNM, for single-cell RNA sequencing. Among these, there were 2 metastatic TDLNs and 6 nonmetastatic TDLNs.

We also generated FFPE [19] samples from the tumor tissue of patients with CRLM at Shanxi Cancer Hospital during surgery, among whom 14 had LNM and 6 did not. In total, there were 20 primary tumors paired with 20 liver metastases, 21 nonmetastatic TDLNs and 12 metastatic TDLNs, for proteomics sequencing. The tumor regions were evaluated by pathological examination.

More detailed methods of sequencing data analysis are presented in the Supplementary Methods.

### Pathological evaluation

We collected tissue samples from CRLM patients at the National Cancer Center during surgery; 24 of these patients had LNM, and 20 did not. The obtained samples comprised 44 primary tumors, 44 paired liver metastases, 51 nonmetastatic TDLNs and 55 metastatic TDLNs. The microsatellite instability (MSI) status of 428 CRLM patients from Shanxi Cancer Hospital was evaluated, and 316 of these patients presented with LNM. All pathological evaluations were conducted by two independent pathologists. In cases of disagreement, a final decision was made by a pathology expert with over 10 years of experience.

H&E staining was used to evaluate the extent of the inflammatory response around the primary tumor and the morphology of the liver metastatic lesion and the TDLNs. For immunohistochemistry (IHC), the densities (cells/mm2) of CD3-, CD8- and CD19-positive immune cells were calculated using image analysis (HALO Indica Labs). Manual counting was performed by an experienced pathologist, and Pearson correlation analysis was conducted between the manual count and the HALO-positive cell count to ascertain the accuracy of machine counting (Supplementary Fig. 1).

The invasion front (IF) that consisted of a width span of 500 μm inside and outside the invasive margin was delineated. The tumor core area (CT) was set as the whole tumor area excluding the 500 μm before the IF. The KM grade was determined as previously described [20]. The invasive margin was analyzed for the presence of immune cells; low-grade tumors exhibited mild or patchy immune cell infiltration without evidence of destruction of cancer cell islets, while high-grade tumors exhibited band-like immune cell infiltration. Crohn-like lymphoid reactions (CLRs) were defined as lymphoid structures surrounding the primary tumors but were excluded if they were (1) associated with the mucous membrane (mucosa-associated lymphoid tissue, MALT); (2) considered parts of mesenteric small lymph nodes; or (3) irregularly shaped, long and narrow, or nonnodular [21]. The number of CLRs was counted, and the length of the invasive front was measured. CLR density was defined as the number of CLR follicles divided by the length of the invasive front [22].

The histological growth patterns (HGPs) of liver metastases are divided into three patterns, namely, the desmoplastic pattern, pushing pattern, and replacement pattern [23]. The specific assessment method has been described in previous studies [24]. Since the desmoplastic pattern is associated with a better prognosis, the pushing pattern and replacement pattern are defined as nondesmoplastic patterns [25]. If multiple HGPs were observed in a single tumor, the pattern observed in more than 50% of the total tumor area was considered the final pattern.

The TDLNs were evaluated based on morphological differences and considered to have germinal center (GC) predominance, lymphocyte predominance, lymphocyte depletion, or other features. The evaluation method has been reported in previous studies [26]. For metastatic TDLNs, pathologists evaluated the remaining lymphoid tissue, excluding the metastatic tumor tissue.

### Statistical analysis

Categorical variables are presented as numbers with percentages, while quantitative variables are presented as medians with interquartile ranges (IQRs), unless indicated otherwise. Student’s *t* test and the Mann‒Whitney *U* test were used for continuous data. Fisher’s exact test was used for categorical data. The log-rank test and Kaplan–Meier survival curve were used to compare CSS between groups. All the statistical tests were 2-sided, and *P* < 0.05 was considered to indicate statistical significance. A significance level of *P* < 0.05 was assumed for all the statistical evaluations. All the statistical analyses and data visualizations were performed using R 4.2.1 software.

More detailed methods of sample preparation and processing are presented in the Supplementary Methods.

## Results

### Baseline characteristics

A total of 8035 and 1305 CRLM patients in the SEER cohort and Chinese cohort, respectively, who underwent surgical resection were included in this study. The baseline characteristics are shown in Supplementary Table 1. We conducted a comparative analysis of baseline characteristics between the LNM group and Non-LNM group, both before and after IPTW, in these two cohorts (Supplementary Table 2). After applying IPTW, the Non-LNM group and the LNM group were similar both in the SEER cohort and the Chinese cohort. This process ensured a well-balanced distribution of baseline characteristics between the two groups, including age at diagnosis, sex, race, primary tumor site, tumor histology type, postoperative radiotherapy and/or chemotherapy, surgical procedure, and tumor size (all *p* > 0.05).

### LNM leads to worse CSS in patients with CRLM

**Fig. 2:**
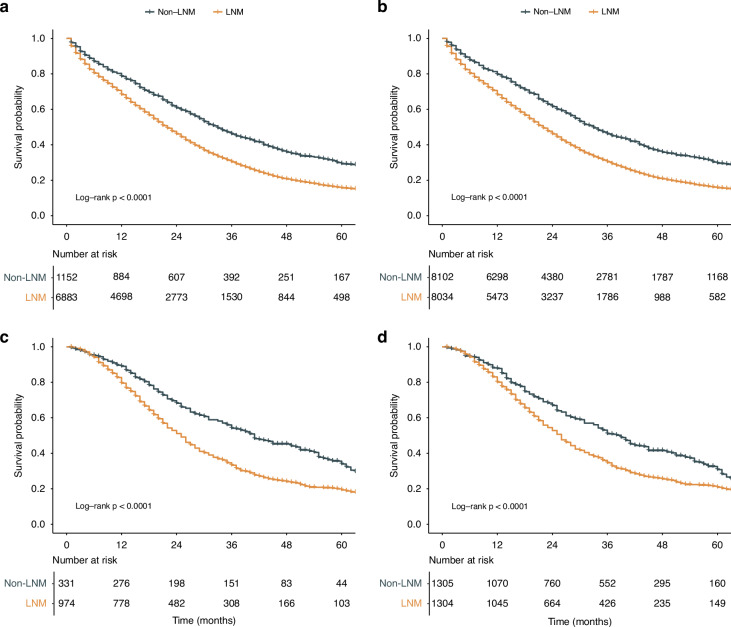
Survival comparisons between CRLM patients with and without LNM. Kaplan‒Meier curve of stratified survival in patients in the SEER cohort: **a** before IPTW, **b** after IPTW. Kaplan‒Meier curve of stratified survival in patients in the Chinese cohort: **c** before IPTW, **d** after IPTW. CRLM colorectal cancer liver metastasis, LNM lymph node metastasis, SEER Surveillance, Epidemiology, and End Results, IPTW inverse probability of treatment weighting.

To further explore the prognostic effect of LNM in patients with CRLM, we conducted Kaplan‒Meier survival analysis. Non-LNM remained associated with a better prognosis even after adjusting for other clinicopathological prognostic factors in the SEER cohort (HR = 1.72; 95% CI, 1.59–1.86; *p* < 0.001), making it an independent prognostic factor for stage IV CRC patients (Supplementary Table 3). The same conclusion was validated in the Chinese cohort (HR = 1.46; 95% CI, 1.24–1.73; *p* < 0.001). Both before and after IPTW, Kaplan‒Meier survival analysis revealed that patients with LNM had significantly worse CSS (all log-rank *p* < 0.001) than patients without LNM in the SEER cohort (Fig. 2a, b). In the Chinese cohort, patients with LNM also had significantly worse CSS (all log-rank *p* < 0.001) than patients without LNM (Fig. 2c, d).

### Gene expression characteristics of metastatic and nonmetastatic TDLNs

The gene expression levels of TDLNs were compared between the metastatic and nonmetastatic TDLN groups. The results revealed that 3116 genes were upregulated, while 1870 genes were downregulated in the metastatic TDLN group (Supplementary Fig. 2a). Among them, the epithelial cell differentiation-related KRT gene family, the cell cycle regulation gene FAM83H, and the tumor metastasis-related genes EPS8L1 and TNS4 were upregulated in metastatic TDLNs. Conversely, immune regulation-related genes such as CLEC4M and CD5L were downregulated in these lymph nodes.

To further characterize the biological attributes distinguishing the metastatic and nonmetastatic TDLN groups, we employed Metascape enrichment analysis. In the metastatic TDLN group, the pathways enriched in the upregulated genes were associated with stromal cell activation, including cell junction and adhesion, fibroblast-mediated extracellular matrix organization, endothelial cell-mediated blood vessel development and epithelial cell differentiation (Supplementary Fig. 2B). The downregulated genes were primarily enriched in pathways related to adaptive immunity, including the regulation of lymphocyte activation, differentiation, immune effector processes and antigen receptor-mediated signaling (Supplementary Fig. 2C).

### Single-cell RNA sequencing reveals differences in the TDLN microenvironment

**Fig. 3:**
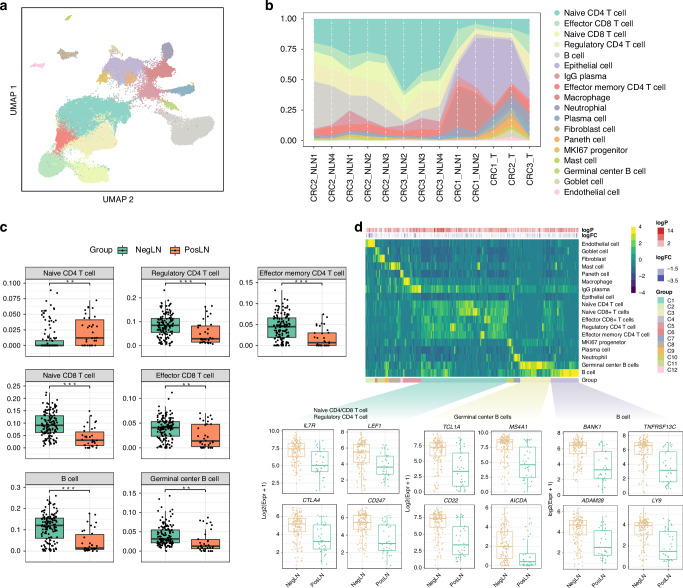
The immune infiltration profile of TDLNs. **a** Uniform manifold approximation and projection (UMAP) analysis of the transcriptional profiles of TDLNs, colored by cell type. **b** Proportion plots of various cell types from different sample sources. **c** Box plots illustrating the differences in immune cell infiltration between negative and positive TDLNs using lymph node sc-RNA sequencing data as a reference based on CIBERSORTx. **d** Heatmap and box plots showing cell types highly expressed DEGs by the expression of upregulated DEGs (PosLN vs. NegLN) across cell types. NegLN negative lymph node, PosLN positive lymph node, TDLNs tumor-draining lymph nodes, sc-RNA single-cell RNA, DEGs differentially expressed genes.

After preprocessing and quality control, our single-cell atlas contained high-quality transcriptomes from 84623 cells spanning both tumor and immune compartments. Unsupervised clustering analysis revealed 18 distinct clusters, including all major known epithelial, mesenchymal, and leukocyte lineages (Fig. 3a). Uniform manifold approximation and projection (UMAP) plots based on the sample of origin further revealed the impact of tumor metastases on the cellular composition of TDLNs (Supplementary Fig. 3A). Compared to nonmetastatic TDLNs, metastatic TDLNs showed a substantial presence of epithelial cells with similar characteristics to those of the primary tumor, consistent with the typical definition of LNM. For accurate cell type determination, we only used highly variable genes between cell types (Supplementary Fig. 3B). We then compared the microenvironmental composition at distinct sites of origin (Fig. 3b). In general, lymphocytes, including CD4^+^ T cells, CD8^+^ T cells, and B cells, represented the largest immune subpopulation within nonmetastatic TDLNs, while metastatic TDLNs contained a relatively greater proportion of fibroblasts, endothelial cells and macrophages.

To further validate the results of single-cell RNA sequencing, we conducted cell composition prediction using bulk RNA sequencing data from the aforementioned 173 TDLNs. Unlike traditional immune infiltration analyses, we conducted cell composition prediction using lymph node single-cell RNA sequencing data as a reference based on CIBERSORTx [27]. The numbers of lymphocytes, including CD4^+^ T cells, CD8^+^ T cells, and B cells, were increased in nonmetastatic TDLNs (Fig. 3c, *p* < 0.001, *p* < 0.001, and *p* < 0.001, respectively). In metastatic TDLNs, there was a significant increase in fibroblasts, epithelial cells, and macrophages (Supplementary Fig. 3C, *p* < 0.05, *p* < 0.001, and *p* < 0.001, respectively). Then, we identified DEGs that were upregulated or downregulated in each cell type by comparing samples from patients with and without LNM. The upregulated DEGs in patients with LNM were also highly expressed in epithelial cells, fibroblasts, macrophages, and endothelial cells (Supplementary Fig. 3D). The downregulated DEGs in patients with LNM were highly expressed in CD4^+^/CD8^+^ T cells and B cells (Fig. 3d).

In addition to changes in cell count and proportion, we further investigated the transcriptomic differences of B cells, CD4+ T cells, CD8+ T cells, and macrophages between metastatic and non-metastatic TDLNs (Supplementary Fig. 4A). First, we performed Gene Ontology (GO)/Kyoto Encyclopedia of Genes and Genomes (KEGG) enrichment analysis on genes downregulated in each cell type in metastatic TDLNs compared to non-metastatic TDLNs. We found that in metastatic TDLNs, biological processes such as B cell activation and B cell-mediated immunity were downregulated, and molecular functions such as immune receptor activity were inhibited (Supplementary Fig. 4B). For both CD4+ T cells and CD8+ T cells, impairments in cell activation and differentiation were found (Supplementary Fig. 4C, D). For macrophages, the biological process of antigen processing and presentation was suppressed, leading to a reduced capacity for producing molecules that mediate immune responses and a diminished ability to regulate adaptive immunity (Supplementary Fig. 4E). Similarly, we also performed enrichment analysis on the upregulated genes, and the results are presented in Supplementary Fig. 5. In conclusion, after the metastasis of tumor cells, the transcriptome of TDLNs is altered to induce immune dysfunction and stromal cell proliferation.

### The impact of LNM on the TDLNs of patients with CRLM

**Fig. 4:**
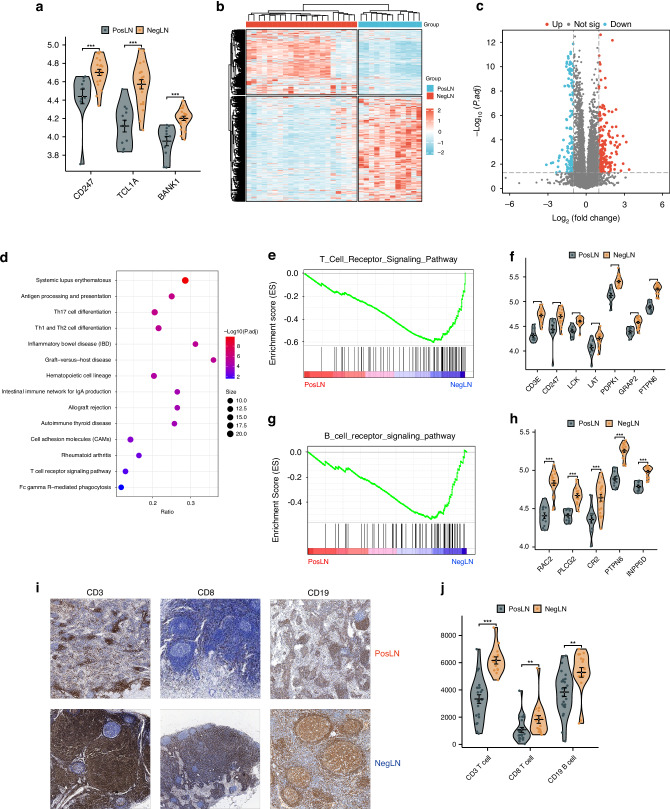
Proteomic and pathological characteristics of positive and negative TDLNs. **a** Violin plots verifying protein differences in immune cell-associated upregulated genes. **b** Heatmap of differentially expressed proteins in positive lymph nodes (PosLNs) and negative lymph nodes (NegLNs); **c** Volcano plot showing differentially expressed proteins in PosLNs and NegLNs; **d** Dot plot showing the KEGG pathways enriched in the proteins upregulated in NegLNs; **e** GSEA plot of the T-cell receptor signaling pathway gene set. **f** Violin plot showing the difference in protein expression between PosLNs and NegLNs in the T-cell receptor signaling pathway gene set. **g** GSEA plot of the B-cell receptor signaling pathway gene set. **h** Violin plot showing the difference in protein expression between PosLNs and NegLNs in the B-cell receptor signaling pathway gene set. **i**, **j** Immunohistochemical staining of CD3, CD8 and CD19 in PosLNs and NegLNs. **i** Pathological images. **j** Violin plot of the quantitative results. PosLN positive lymph node, NegLN negative lymph node, TDLNs tumor-draining lymph nodes, KEGG Kyoto Encyclopedia of Genes and Genomes, GSEA gene set enrichment analysis.

We first validated the above findings via proteomic sequencing. We found that the T-cell marker gene CD247 and the B-cell marker genes TCL1A and BANK1 were upregulated at the protein level, while the epithelial cell marker gene MACC1 and the macrophage marker genes FBP1, SPP1, and TFRC were downregulated (Fig. 4a and Supplementary Fig. 6A). A total of 309 proteins were differentially expressed, with 187 upregulated and 122 downregulated (Fig. 4b, c). To further explore the biological processes associated with LNM in the TDLNs of patients with CRLM, we conducted pathway enrichment analysis. KEGG enrichment analysis revealed that the downregulated proteins were mainly associated with immune-related pathways, including the T/B-cell receptor signaling pathway, helper T-cell differentiation pathway, and antigen processing and presentation pathway (Fig. 4d). GSEA also demonstrated the same results (Fig. 4e, g, Supplementary Fig. 6B, C). We also examined the expression of proteins related to these four downregulated pathways and found that they were all downregulated in metastatic TDLNs (Fig. 4f, h, Supplementary Fig. 6D). Additionally, we found that the upregulated proteins were mainly enriched in the extracellular matrix (ECM)-receptor interaction pathway, with ECM-related proteins such as COL1A1, FN1, and ITGB3 being upregulated in metastatic TDLNs (Supplementary Fig. 6E–G).

We further validated the impact of LNM on the immune function of TDLNs at the pathological level. When LNM occurred, the immune microenvironment of the TDLNs was comprehensively suppressed, leading to a decrease in the density of CD3^+^ T cells, CD8^+^ T cells, and CD19^+^ B cells (Fig. 4i, j, *p* < 0.001, *p* = 0.031, and *p* < 0.001, respectively). Morphologically, metastatic TDLNs were predominantly characterized by the lymphocyte depletion type (Supplementary Fig. 7, 36.4% vs. 9.8%, *p* < 0.05), while nonmetastatic TDLNs were predominantly characterized by the GC predominance type (41.2% vs. 14.5%, *p* < 0.05). No significant differences were observed between the LNM group and Non-LNM group in terms of lymphocyte predominance type (36.4% vs. 41.2%, *p* > 0.05). In summary, our proteomic and pathological analyses both revealed immune dysfunction in metastatic TDLNs.

### The impact of LNM on primary tumors in patients with CRLM

Next, we validated the impact of LNM on CRLM at the level of the primary tumor. Only 52 proteins were differentially expressed in the primary tumor, with 33 upregulated and 19 downregulated DEGs (Supplementary Fig. 8A, B). GO enrichment analysis indicated that the downregulated proteins were mainly associated with immune response-related biological processes (Supplementary Fig. 8C, D). Unfortunately, we did not find any pathways enriched in differentially expressed proteins based on the KEGG enrichment analysis.

**Fig. 5:**
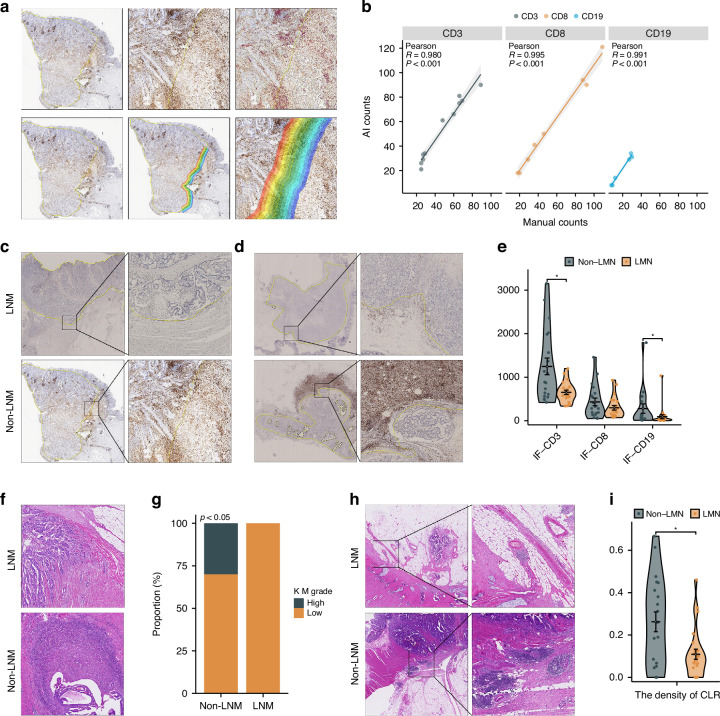
Pathological characteristics of primary tumors with and without LNM. **a** Halo image analysis automatically identified IHC-positive cells at the IF. **b** Pearson correlation analysis between manual counting and AI counting, showing the accuracy of Halo-image analysis. IHC staining of CD3 and CD19 at the IF of primary tumors with and without LNM: **c** CD3, **d** CD19. **e** Violin plot showing the quantitative results of IHC staining. Differences in the KM grade of primary tumors with and without LNM: **f** pathological images, **g** bar plot of quantitative results. Differences in CLR count between primary tumors with and without LNM: **h** pathological images, **i** violin plot of the quantitative results. AI artificial intelligence, LNM lymph node metastasis, IF invasive front, KM Klintrup–Makinen, CLR Crohn-like lymphoid reaction, IHC immunohistochemistry.

However, we did observe the impact of LNM on the primary tumor at the pathological level. Halo image analysis was applied to accurately assess the number of IF-positive cells according to Pearson correlation analysis (Fig. 5a, b, *p* < 0.001). Compared to that of those in the group without LNM, the density of CD3^+^ T cells and CD19^+^ B cells in the IF in the group with LNM was significantly lower (Fig. 5c–e, *p* = 0.018 and *p* = 0.017, respectively). Moreover, 30% of the primary tumors had a high KM grade in the non-LNM group, which was significantly greater than the proportion of tumors with a high KM grade in the LNM group (Fig. 5f, g, *p* < 0.05). The density and number of CLRs were greater in the group without LNM (Fig. 5h, i and Supplementary Fig. 8F, *p* = 0.006 and *p* = 0.038, respectively). The group without LNM had a greater proportion of patients with deficient mismatch repair (dMMR) status (Supplementary Fig. 8H, 10.7% vs. 4.1%, *p* < 0.05). We further investigated the prognostic impact of LNM under different MSI statuses in CRLM patients. Patients with pMMR without LNM had a better prognosis (Supplementary Fig. 9A, log-rank *p* < 0.0001), whereas among dMMR patients, there was no statistically significant difference in prognosis between those with and without LNM (Supplementary Fig. 9B, log-rank *p* = 0.64). However, the LNM group showed a trend toward poorer outcomes. No significant differences were observed between the two groups in the other subgroup analyses (Fig. 5e and Supplementary Fig. 8). In conclusion, we found that the TDLN status affected the immune status of the primary tumor, although this effect was spatially localized.

### The impact of LNM on liver metastases in patients with CRLM

**Fig. 6:**
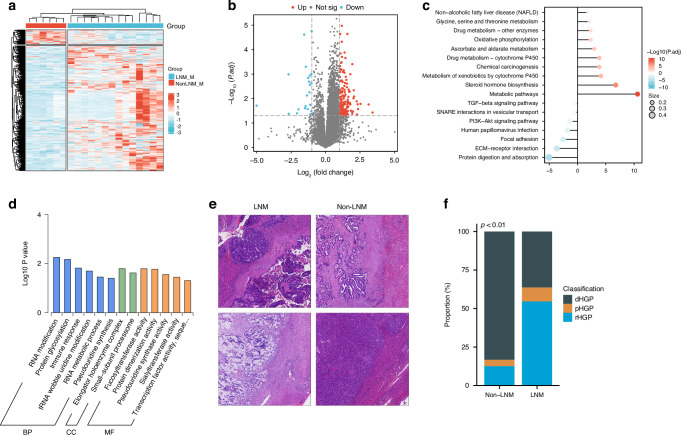
Proteomic and pathological characteristics of liver metastasis with and without LNM. **a** Heatmap of differentially expressed proteins in liver metastasis with and without LNM. **b** Volcano plot showing differentially expressed proteins in liver metastasis with and without LNM. **c** Lollipop diagram illustrating the KEGG pathways enriched in the proteins upregulated and downregulated in liver metastases. **d** Bar plot illustrating the GO terms enriched in proteins downregulated in samples from patients with liver metastasis with LNM; **e**, **f** Differences in the HGP of samples from patients with liver metastasis with and without LNM: **e** pathological images, **f** bar plot of quantitative results. BP biological process, CC cellular component, MF molecular function, LNM lymph node metastasis, HGP histological growth pattern, d desmoplastic, p pushing, r replacement, KEGG Kyoto Encyclopedia of Genes and Genomes, GO Gene Ontology.

Finally, we further investigated the impact of LNM on liver metastasis. A total of 190 proteins were differentially expressed, with 168 upregulated and 22 downregulated DEGs (Fig. 6a, b). KEGG enrichment analysis revealed that the upregulated proteins were mainly enriched in metabolic pathways, including drug metabolism, protein metabolism, and oxidative phosphorylation, while the downregulated proteins were primarily associated with cell proliferation-related pathways, such as the TGF-β signaling pathway and the PI3K-A signaling pathway (Fig. 6c). The GO enrichment analysis also indicated that the downregulated proteins were mainly associated with immune response-related biological processes (Fig. 6d).

Although we found no significant difference in the density of immune cells in liver metastatic lesions between the two groups (Supplementary Fig. 10), the morphological evaluation revealed that the HGP of liver metastatic lesions was predominantly desmoplastic HGP (dHGP) in the group without LNM, while it was predominantly a non-dHGP, including the pushing HGP (pHGP) and the replacement HGP (rHGP), in the group with LNM (Fig. 6e, f, *p* < 0.01).

## Discussion

CRC is the second leading cause of cancer-related mortality worldwide, with the liver being the most frequent site of distant metastasis. However, the metastasis status of TDLNs in patients with CRLM has not been adequately studied. In this study, we first validated that CRLM patients with LNM had a worse prognosis than those without LNM. Furthermore, at the transcriptomic, proteomic, and histopathological levels, we found that LNM inhibits the immune activity of TDLNs in CRLM, reduces the immune and inflammatory functions of the primary tumor, and affects the histological growth pattern of liver metastases. Finally, based on two large population-based cohorts and integrated multiomics resources, for the first time, we revealed how LNM adversely affects the prognosis of CRLM. These findings highlight the potential value of LNM status as a crucial aspect for refining the staging of CRLM.

TDLNs primarily participate in tumor immunity through tumor antigen presentation and recognition, activation and infiltration of immune cells, and immune surveillance. The normal functioning of the immune system is crucial for effectively clearing tumor cells [28]. However, due to the highly invasive nature of tumor cells, during disease progression, tumor cells can metastasize to TDLNs via lymphatic pathways, thereby affecting the immune function of TDLNs [10]. The specific reasons can be summarized as follows: First, TDLNs primarily exert their immune function through the lymphatic circulation, and tumor metastasis can obstruct lymphatic vessels, thereby affecting the immune function of lymph nodes. Second, tumor cells within TDLNs can occupy space and compress normal lymphatic tissue, leading to anatomical damage to the TDLNs. Third, the disruption of the normal TDLN structure and the release of immunosuppressive factors by tumor cells reduce the activity and quantity of immune cells within the TDLNs. Therefore, LNM is widely recognized as a significant risk factor for various solid tumors [29–31]. Based on two large multicenter cohorts, we validated that LNM remains an independent risk factor even for CRC patients who have developed distant metastasis. As reported by S. Ahmed et al., LNM is an important prognostic factor independent of chemotherapy and metastasectomy in stage IV CRC patients [32]. Compared to previous studies, our study leveraged a large sample size and employed IPTW to reduce intergroup confounding biases, thus enhancing the rigor and reliability of our findings.

To further elucidate the mechanisms underlying the impact of LNM on the prognosis of patients with CRLM, we first investigated the effect of LNM on TDLNs. Based on bulk RNA sequencing of 173 TDLNs, we explored the transcriptomic characteristics of metastatic and nonmetastatic LNs and revealed a significant association between the enrichment of immune activation-related pathways and the presence of nonmetastatic LNs. In contrast, the presence of metastatic LNs was significantly associated with the enrichment of pathways related to matrix proliferation, particularly fibroblast-mediated extracellular matrix formation, which may lead to LN fibrosis. To clearly describe the cellular-level changes caused by tumor cell (mainly epithelial cells) invasion into LNs, we performed single-cell RNA sequencing of TDLNs. Additionally, to address the limitations of the small sample size in single-cell sequencing, we innovatively utilized TDLN single-cell sequencing data as a reference to conduct immune infiltration prediction analysis on the bulk RNA sequencing data of 173 TDLNs. Based on these two transcriptional datasets, we found that nonmetastatic TDLNs harbored a greater abundance of lymphocytes, including CD4^+^ T cells, CD8^+^ T cells, and CD19^+^ B cells, representing the largest immune subpopulation. In contrast, metastatic TDLNs contained a relatively greater proportion of fibroblasts, endothelial cells, and macrophages. Considering that tumor cell invasion inevitably alters the proportion of each cell type within metastatic TDLNs, we further analyzed the functional changes in each cell type to better characterize the immune microenvironment of TDLNs. We found that in metastatic TDLNs, both B and T cells showed not only a decrease in number and proportion but also downregulation of key functions, including cell activation, cell-mediated immune responses, and differentiation, while pathways such as apoptosis were upregulated. This suggests that tumor cells may reduce immune cell counts by disrupting the TDLNs’ structure. Additionally, tumor cells may interfere with immune cell function through direct contact or by producing mediators. Further research is needed to explore the underlying mechanisms of these hypotheses.

Previous studies have reported that an intact LN structure can enhance the response of CD8^+^ T cells to immunotherapy [15]. Therefore, the changes in the number of immune cells caused by LNM may be partly due to the disruption of normal LN structure by tumor cells. However, it is currently unclear whether the remaining normal lymphatic tissue in metastatic LNs also undergoes immune suppression due to invasion by tumor cells. To address this issue, we further explored proteomic sequencing and histopathological staining in two cohorts of patients with CRLM. The proteomic data validated our conclusions regarding changes in cell numbers and reaffirmed the downregulation of immune function in metastatic TDLNs at the gene expression level. Immune-related pathways such as T/B-cell receptor signaling, helper T-cell differentiation, and antigen presentation were downregulated. IHC staining further supported the above results, showing that in CRLM patients, the numbers of CD3^+^ T cells, CD8^+^ T cells, and CD19^+^ B cells in nonmetastatic TDLNs were greater than those in metastatic TDLN normal lymphatic tissue. Additionally, H&E staining of slides revealed that CRLM patients without LNM (T_any_N_-_M_1_) had more GC-predominant TDLNs, while those with LNM (T_any_N_+_M_1_) had more lymphocyte-depleted TDLNs. In conclusion, we found that for patients with CRLM, LNM ultimately leads to stromal proliferation and a decrease in antitumor immune function in TDLNs.

The antitumor immune response is highly dynamic and truly systemic and involves interactions between the primary tumor, peripheral circulation, and TDLNs [33]. Lal et al. identified a strong correlation between an activated immune response in primary tumors and a high LNs yield [34]. Therefore, we further investigated whether TDLN dysfunction caused by tumor metastasis affects the primary tumor. Proteomic sequencing did not reveal significant differences in protein expression between primary tumors with and without LNM. However, we believe that this may be attributed to tumor heterogeneity, especially intratumoral spatial heterogeneity [35, 36]. Therefore, our research directly assessed pathological characteristics. In our analysis of H&E-stained slides, we found that primary tumors without LNM had a greater proportion of tumors with high-grade KM scores than primary tumors with LNM, suggesting a more pronounced inflammatory response at the IF of the tumor. M. Climent et al. reported that the KM grade reflects the host’s immune response to the tumor and has prognostic value [20]. A higher KM grade is associated with a better prognosis in CRC patients [37]. IHC staining also validated this conclusion, as the IF of primary tumors without LNM revealed increased numbers of CD3^+^ T cells and CD19^+^ B cells.

Additionally, we detected a greater number and greater density of CLRs, namely, tertiary lymphoid structures (TLSs), in these primary tumors. TLSs are lymph node-like structures formed within nonlymphoid tissues, such as the tumor microenvironment, reflecting enhanced local immune responses [38]. The presence of TLSs is associated with a better clinical prognosis across various solid tumors [39–41]. To our knowledge, this study is the first to reveal the significant influence of TDLNs on the formation of tertiary lymphoid structures within primary tumors. Further in-depth investigation into the mechanisms underlying this phenomenon is needed in the future.

CRC patients with dMMR are characterized by hypermutation, resulting in abundant neoantigens that activate an antitumor immune response within the tumor microenvironment [42]. Interestingly, we found a higher dMMR rate in primary tumors without LNM. This suggests a potential link between the immune status of the primary tumor, MSI status, and LNM. Furthermore, we explored the prognostic impact of LNM across different MSI statuses. We observed that pMMR patients without LNM had a better prognosis, whereas in dMMR patients, there was no statistically significant difference in prognosis between those with and without LNM, possibly due to the limited sample size in the dMMR group. However, the LNM group showed a trend toward poorer outcomes. In summary, we found that primary tumors without LNM exhibit enhanced antitumor immune capability.

The primary characteristic of stage IV CRC is the occurrence of distant organ metastasis, predominantly in the liver. Naxerova et al. found that in 65% of cases, lymphatic and distant metastases originated from independent subclones within the primary tumor, while in 35% of cases, they shared a common subclonal origin [43]. This indicates that there are two distinct lineage relationships between lymphatic and distant metastases in CRC. However, due to the considerable distance between TDLNs and liver metastases in terms of anatomical location, as well as the differences in tumor metastasis pathways, there are currently no studies reporting whether TDLN metastasis affects liver metastases. Therefore, we also investigated the immune infiltration status of liver metastases between the LNM group and the Non-LNM group based on pathological IHC staining. Unfortunately, no significant differences were found in the abundance of CD3^+^ T cells, CD8^+^ T cells, or CD19^+^ B cells in the CT or IF images of liver metastases between the two groups based on IHC staining. According to the findings reported by S. Nanji et al., patients with resectable CRC liver metastases with hepatic LN involvement have inferior survival compared to patients with negative nodes [44]. This may suggest that the antitumor immune function of liver metastases in stage IV CRC patients is primarily influenced by the liver regional LNs rather than the TDLNs of the primary tumor. Interestingly, we detected differences in the growth patterns of liver metastatic lesions between the two groups. The liver metastases in the Non-LNM group were predominantly of the dHGP, while those in the LNM group were predominantly of the rHGP. The HGP classification system, proposed by Kudo et al. in 2013, mainly includes dHGP, rHGP, and pHGP. In the dHGP subtype, liver metastases exhibit a distinct fibrous capsule, with clear tumor boundaries. In contrast, liver metastases of the rHGP subtype have unclear boundaries. Previous studies have indicated that the dHGP subtype is typically associated with a better prognosis, while non-dHGP subtype lesions are associated with a poorer prognosis [24, 25]. This may be attributed to the clear tumor boundaries and intact fibrous capsule, which are more favorable for treatments such as surgical resection. Proteomic sequencing revealed that liver metastases without LNM exhibited downregulation of cell proliferation-related pathways, such as the TGF-β and PI3K-AKT pathways. This finding may help explain the mechanism by which TDLNs of the primary tumor affect the growth pattern of liver metastatic lesions. However, further research is needed. Nonetheless, we have provided a novel perspective on the poorer prognosis of CRC with liver metastases and LNM.

Despite these remarkable findings, we would like to acknowledge some of the potential limitations of our study. First, the tumor microenvironment is dynamic. Although our study represents the largest investigation to date into the impact of tumor metastasis on TDLNs using transcriptomic data, its characterization based on transcriptomic data obtained from these samples at a snapshot in time will not comprehensively address spatiotemporal heterogeneity. We hope that future efforts to leverage single-cell spatial transcriptomic data will provide more valuable insights. Second, it is important to acknowledge that our research is a retrospective case‒control study and does not directly establish causal relationships between LNM and many intergroup differences. Nevertheless, we believe this study is highly valuable because it provides initial evidence and valuable insights into the poorer prognosis following LNM in CRLM patients for the first time, laying important groundwork for further in-depth investigation. Finally, we did not investigate whether LNM affects therapeutic options for CRLM patients, particularly regarding immunotherapy and adjuvant chemotherapy. Further exploration through rigorous prospective randomized controlled cohorts is needed to elucidate these findings.

In summary, based on two large population-based cohorts, we confirmed the adverse prognostic impact of LNM on patients with CRLM and, for the first time, explored potential mechanisms through multiomics resources. Specifically, tumor cell invasion leads to immune dysfunction in TDLNs, further reciprocally inhibiting antitumor immune function at the primary tumor site and altering the growth pattern of liver metastases. In clinical practice, our research paves the way for further refinement of the AJCC TNM staging system for CRLM.

## Supplementary information

Supplementary Materials

## Data Availability

The raw CRC patients’ information used in this paper are available from the lead contact upon request. RNA-seq and IHC data are available from the authors on reasonable request and approval of data sharing by institutional review boards. Correspondence for materials should be addressed to the lead contact. Any additional information required to reanalyze the data reported in this paper is available from the lead contact upon request.

## References

[1] Sung H, Ferlay J, Siegel RL, Laversanne M, Soerjomataram I, Jemal A, et al. Global Cancer Statistics 2020: GLOBOCAN Estimates of Incidence and Mortality Worldwide for 36 Cancers in 185 Countries. CA Cancer J Clin. 2021;71:209–49. 33538338 10.3322/caac.21660

[2] Tauriello DV, Calon A, Lonardo E, Batlle E. Determinants of metastatic competency in colorectal cancer. Mol Oncol. 2017;11:97–119. 28085225 10.1002/1878-0261.12018 PMC5423222

[3] Rees M, Tekkis PP, Welsh FK, O’Rourke T, John TG. Evaluation of long-term survival after hepatic resection for metastatic colorectal cancer: a multifactorial model of 929 patients. Ann Surg. 2008;247:125–35. 18156932 10.1097/SLA.0b013e31815aa2c2

[4] Seeberg LT, Brunborg C, Waage A, Hugenschmidt H, Renolen A, Stav I, et al. Survival Impact of Primary Tumor Lymph Node Status and Circulating Tumor Cells in Patients with Colorectal Liver Metastases. Ann Surg Oncol. 2017;24:2113–21. 28258416 10.1245/s10434-017-5818-2 PMC5491630

[5] Dekker E, Tanis PJ, Vleugels JLA, Kasi PM, Wallace MB. Colorectal cancer. Lancet. 2019;394:1467–80. 31631858 10.1016/S0140-6736(19)32319-0

[6] Locker GY, Hamilton S, Harris J, Jessup JM, Kemeny N, Macdonald JS, et al. ASCO 2006 update of recommendations for the use of tumor markers in gastrointestinal cancer. J Clin Oncol. 2006;24:5313–27. 17060676 10.1200/JCO.2006.08.2644

[7] Amin MB, Greene FL, Edge SB, Compton CC, Gershenwald JE, Brookland RK, et al. The Eighth Edition AJCC Cancer Staging Manual: Continuing to build a bridge from a population-based to a more “personalized” approach to cancer staging. CA Cancer J Clin. 2017;67:93–9. 28094848 10.3322/caac.21388

[8] Esterházy D, Canesso MCC, Mesin L, Muller PA, de Castro TBR, Lockhart A, et al. Compartmentalized gut lymph node drainage dictates adaptive immune responses. Nature. 2019;569:126–30. 30988509 10.1038/s41586-019-1125-3 PMC6587593

[9] Dammeijer F, van Gulijk M, Mulder EE, Lukkes M, Klaase L, van den Bosch T, et al. The PD-1/PD-L1-Checkpoint Restrains T cell Immunity in Tumor-Draining Lymph Nodes. Cancer Cell. 2020;38:685–700.e8. 33007259 10.1016/j.ccell.2020.09.001

[10] du Bois H, Heim TA, Lund AW. Tumor-draining lymph nodes: At the crossroads of metastasis and immunity. Sci Immunol. 2021;6:eabg3551. 34516744 10.1126/sciimmunol.abg3551 PMC8628268

[11] Grant SM, Lou M, Yao L, Germain RN, Radtke AJ. The lymph node at a glance - how spatial organization optimizes the immune response. J Cell Sci. 2020;133:jcs241828. 10.1242/jcs.241828 PMC7063836 32144196

[12] Förster R, Braun A, Worbs T. Lymph node homing of T cells and dendritic cells via afferent lymphatics. Trends Immunol. 2012;33:271–80. 22459312 10.1016/j.it.2012.02.007

[13] Mellman I, Chen DS, Powles T, Turley SJ. The cancer-immunity cycle: Indication, genotype, and immunotype. Immunity. 2023;56:2188–205. 37820582 10.1016/j.immuni.2023.09.011

[14] Huang Q, Wu X, Wang Z, Chen X, Wang L, Lu Y, et al. The primordial differentiation of tumor-specific memory CD8(+) T cells as bona fide responders to PD-1/PD-L1 blockade in draining lymph nodes. Cell. 2022;185:4049–66.e25. 10.1016/j.cell.2022.09.020 36208623

[15] Rahim MK, Okholm TLH, Jones KB, McCarthy EE, Liu CC, Yee JL, et al. Dynamic CD8(+) T cell responses to cancer immunotherapy in human regional lymph nodes are disrupted in metastatic lymph nodes. Cell. 2023;186:1127–43.e18. 36931243 10.1016/j.cell.2023.02.021 PMC10348701

[16] Reticker-Flynn NE, Engleman EG. Lymph nodes: at the intersection of cancer treatment and progression. Trends Cell Biol. 2023;33:1021–34. 37149414 10.1016/j.tcb.2023.04.001 PMC10624650

[17] Thomas L, Li F, Pencina M. Using Propensity Score Methods to Create Target Populations in Observational Clinical Research. JAMA. 2020;323:466–7. 31922529 10.1001/jama.2019.21558

[18] Doll KM, Rademaker A, Sosa JA. Practical Guide to Surgical Data Sets: Surveillance, Epidemiology, and End Results (SEER) Database. JAMA Surg 2018;153:588–9. 29617544 10.1001/jamasurg.2018.0501

[19] Buczak K, Kirkpatrick JM, Truckenmueller F, Santinha D, Ferreira L, Roessler S, et al. Spatially resolved analysis of FFPE tissue proteomes by quantitative mass spectrometry. Nat Protoc. 2020;15:2956–79. 32737464 10.1038/s41596-020-0356-y

[20] Feliciano EMC, Kroenke CH, Meyerhardt JA, Prado CM, Bradshaw PT, Kwan ML, et al. Association of Systemic Inflammation and Sarcopenia With Survival in Nonmetastatic Colorectal Cancer: Results From the C SCANS Study. JAMA Oncol. 2017;3:e172319. 28796857 10.1001/jamaoncol.2017.2319 PMC5824285

[21] Ueno H, Hashiguchi Y, Shimazaki H, Shinto E, Kajiwara Y, Nakanishi K, et al. Objective criteria for crohn-like lymphoid reaction in colorectal cancer. Am J Clin Pathol. 2013;139:434–41. 23525613 10.1309/AJCPWHUEFTGBWKE4

[22] Väyrynen JP, Sajanti SA, Klintrup K, Mäkelä J, Herzig KH, Karttunen TJ, et al. Characteristics and significance of colorectal cancer associated lymphoid reaction. Int J Cancer. 2014;134:2126–35. 24154855 10.1002/ijc.28533

[23] Vermeulen PB, Colpaert C, Salgado R, Royers R, Hellemans H, Van Den Heuvel E, et al. Liver metastases from colorectal adenocarcinomas grow in three patterns with different angiogenesis and desmoplasia. J Pathol. 2001;195:336–42. 11673831 10.1002/path.966

[24] Nielsen K, Rolff HC, Eefsen RL, Vainer B. The morphological growth patterns of colorectal liver metastases are prognostic for overall survival. Mod Pathol. 2014;27:1641–8. 24851832 10.1038/modpathol.2014.4

[25] Oliveira RC, Alexandrino H, Cipriano MA, Alves FC, Tralhão JG. Predicting liver metastases growth patterns: Current status and future possibilities. Semin Cancer Biol. 2021;71:42–51. 32679190 10.1016/j.semcancer.2020.07.007

[26] Tsakraklides V, Wanebo HJ, Sternberg SS, Stearns M, Good RA. Prognostic evaluation of regional lymph node morphology colorectal cancer. Am J Surg. 1975;129:174–80. 1119677 10.1016/0002-9610(75)90294-9

[27] Newman AM, Liu CL, Green MR, Gentles AJ, Feng W, Xu Y, et al. Robust enumeration of cell subsets from tissue expression profiles. Nat Methods. 2015;12:453–7. 25822800 10.1038/nmeth.3337 PMC4739640

[28] Grant SM, Lou M, Yao L, Germain RN, Radtke AJ, Lennon-Duménil A-M. The lymph node at a glance – how spatial organization optimizes the immune response. J Cell Sci. 2020;133:jcs241828. 10.1242/jcs.241828 PMC7063836 32144196

[29] de Boer M, van Dijck JA, Bult P, Borm GF, Tjan-Heijnen VC. Breast cancer prognosis and occult lymph node metastases, isolated tumor cells, and micrometastases. J Natl Cancer Inst. 2010;102:410–25. 20190185 10.1093/jnci/djq008

[30] Kayani B, Zacharakis E, Ahmed K, Hanna GB. Lymph node metastases and prognosis in oesophageal carcinoma-a systematic review. Eur J Surg Oncol. 2011;37:747–53. 21839394 10.1016/j.ejso.2011.06.018

[31] Zumsteg ZS, Luu M, Kim S, Tighiouart M, Mita A, Scher KS, et al. Quantitative lymph node burden as a ‘very-high-risk’ factor identifying head and neck cancer patients benefiting from postoperative chemoradiation. Ann Oncol. 2019;30:76–84. 30395159 10.1093/annonc/mdy490 PMC6336000

[32] Ahmed S, Leis A, Chandra-Kanthan S, Fields A, Zaidi A, Abbas T, et al. Regional Lymph Nodes Status and Ratio of Metastatic to Examined Lymph Nodes Correlate with Survival in Stage IV Colorectal Cancer. Ann Surg Oncol. 2016;23:2287–94. 27016291 10.1245/s10434-016-5200-9

[33] Wall I, Boulat V, Shah A, Blenman KRM, Wu Y, Alberts E, et al. Leveraging the Dynamic Immune Environment Triad in Patients with Breast Cancer: Tumour, Lymph Node, and Peripheral Blood. Cancers. 2022;14:4505. 10.3390/cancers14184505 PMC9496983 36139665

[34] Lal N, Chan DKH, Ng ME, Vermeulen L, Buczacki SJA. Primary tumour immune response and lymph node yields in colon cancer. Br J Cancer. 2022;126:1178–85. 35043009 10.1038/s41416-022-01700-1 PMC9023574

[35] Wu L, Yan J, Bai Y, Chen F, Zou X, Xu J, et al. An invasive zone in human liver cancer identified by Stereo-seq promotes hepatocyte-tumor cell crosstalk, local immunosuppression and tumor progression. Cell Res. 2023;33:585–603. 37337030 10.1038/s41422-023-00831-1 PMC10397313

[36] Ye Y, Wu X, Wang H, Ye H, Zhao K, Yao S, et al. Artificial intelligence-assisted analysis for tumor-immune interaction within the invasive margin of colorectal cancer. Ann Med. 2023;55:2215541. 37224471 10.1080/07853890.2023.2215541 PMC10210840

[37] Climent M, Ryan ÉJ, Stakelum Á, Khaw YL, Creavin B, Lloyd A, et al. Systemic inflammatory response predicts oncological outcomes in patients undergoing elective surgery for mismatch repair-deficient colorectal cancer. Int J Colorectal Dis. 2019;34:1069–78. 30993458 10.1007/s00384-019-03274-6

[38] Schumacher TN, Thommen DS. Tertiary lymphoid structures in cancer. Science. 2022;375:eabf9419. 34990248 10.1126/science.abf9419

[39] Zhang C, Wang XY, Zuo JL, Wang XF, Feng XW, Zhang B, et al. Localization and density of tertiary lymphoid structures associate with molecular subtype and clinical outcome in colorectal cancer liver metastases. J Immunother Cancer. 2023;11:e006425. 10.1136/jitc-2022-006425 PMC9923349 36759015

[40] He M, He Q, Cai X, Liu J, Deng H, Li F, et al. Intratumoral tertiary lymphoid structure (TLS) maturation is influenced by draining lymph nodes of lung cancer. J Immunother Cancer. 2023;11:e005539. 10.1136/jitc-2022-005539 PMC10124324 37072348

[41] Jia W, Yao Q, Wang Y, Mao Z, Zhang T, Li J, et al. Protective effect of tertiary lymphoid structures against hepatocellular carcinoma: New findings from a genetic perspective. Front Immunol. 2022;13:1007426. 36189217 10.3389/fimmu.2022.1007426 PMC9515394

[42] Jin Z, Sinicrope FA. Mismatch Repair-Deficient Colorectal Cancer: Building on Checkpoint Blockade. J Clin Oncol. 2022;40:2735–50. 35649217 10.1200/JCO.21.02691 PMC9390830

[43] Naxerova K, Reiter JG, Brachtel E, Lennerz JK, van de Wetering M, Rowan A, et al. Origins of lymphatic and distant metastases in human colorectal cancer. Science. 2017;357:55–60. 28684519 10.1126/science.aai8515 PMC5536201

[44] Nanji S, Tsang ME, Wei X, Booth CM. Regional lymph node involvement in patients undergoing liver resection for colorectal cancer metastases. Eur J Surg Oncol. 2017;43:322–9. 28057391 10.1016/j.ejso.2016.10.033

