# Correction: Impact of lymph node metastasis on immune microenvironment and prognosis in colorectal cancer liver metastasis: insights from multiomics profiling

**DOI:** 10.1038/s41416-025-02941-6

**Published:** 2025-02-17

**Authors:** Yueyang Zhang, Deng Wu, Zhen Zhang, Jian Ma, Shuai Jiao, Xiaolong Ma, Jiangtao Li, Yongsheng Meng, Zhixun Zhao, Haipeng Chen, Zheng Jiang, Guiyu Wang, Haiyi Liu, Yanfeng Xi, Haitao Zhou, Xishan Wang, Xu Guan

**Affiliations:** 1 Department of Colorectal Surgery, National Cancer Center/National Clinical Research Center for Cancer/Cancer Hospital, Chinese Academy of Medical Sciences and Peking Union Medical College, Beijing, China; 2 College of Biomedical Information and Engineering, Hainan Medical University, Haikou, China; 3 Department of Pathology, Shanxi Province Cancer Hospital/Shanxi Hospital Affiliated to Cancer Hospital, Chinese Academy of Medical Sciences/Cancer Hospital Affiliated to Shanxi Medical University, Taiyuan, China; 4 Department of Colorectal Surgery, Shanxi Province Cancer Hospital/Hospital Affiliated to Cancer Hospital, Chinese Academy of Medical Sciences/Cancer Hospital Affiliated to Shanxi Medical University, Taiyuan, China; 5 Department of Colorectal Surgery, Nanjing Drum Tower Hospital, the Affiliated Hospital of Nanjing University Medical School, Nanjing, China; 6 Department of Pathology, National Cancer Center/National Clinical Research Center for Cancer/Cancer Hospital, Chinese Academy of Medical Sciences and Peking Union Medical College, Beijing, China; 7 Department of Tumor Biobank, Shanxi Province Cancer Hospital/ Shanxi Hospital Affiliated to Cancer Hospital, Chinese Academy of Medical Sciences/ Cancer Hospital Affiliated to Shanxi Medical University, Taiyuan, China; 8 Department of Colorectal Cancer Surgery, the Second Affiliated Hospital of Harbin Medical University, 246 Xuefu Road, Nangang District Harbin, China

**Keywords:** Metastasis, Colorectal cancer

Correction to: *British Journal of Cancer* 10.1038/s41416-024-02921-2, published online 03 January 2025

During the production process, an error was introduced in Fig. 5 (panels 5C and 5D were duplicated). Panel 5D has been reverted to the version seen by the journal editors and reviewers during the peer review process.


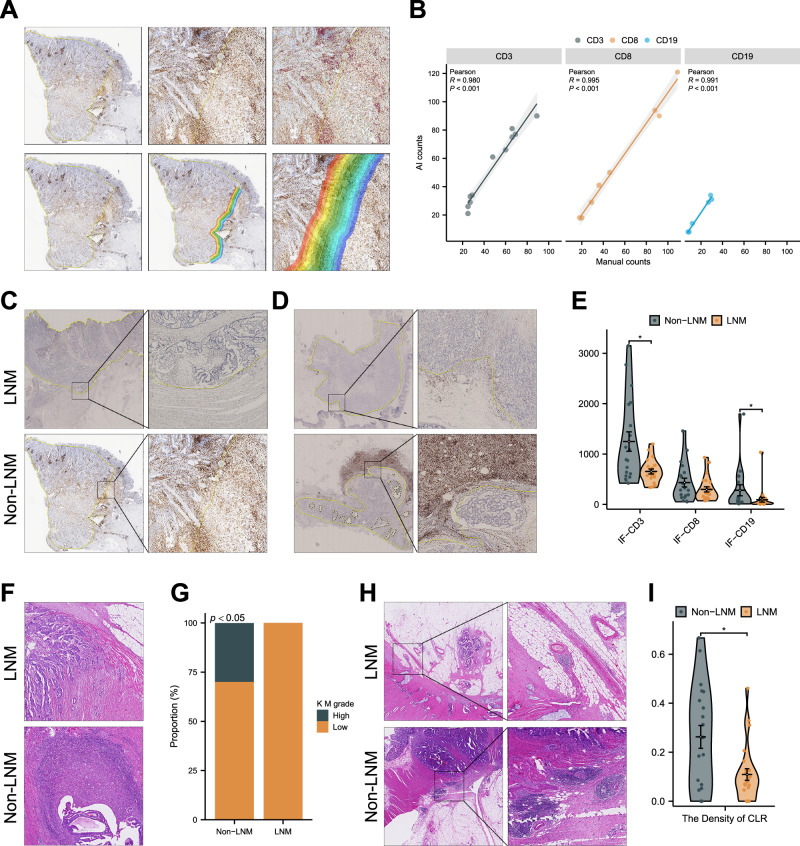


The original article has been updated.

