# Supplementary material for: Impact of lymph node metastasis on immune microenvironment and prognosis in colorectal cancer liver metastasis: insights from multiomics profiling
Source: Br J Cancer. 2025 Jan 3;132(6):513–24. doi: 10.1038/s41416-024-02921-2 (PMC11920064; doi:10.1038/s41416-024-02921-2)
Supplement: Supplementary file 1 — Supplementary Materials [file 41416_2024_2921_MOESM1_ESM.pdf]

Supplementary methods

Bulk RNA sequencing sample preparation and processing

We extracted RNA from Fresh-frozen specimens using TRIzol reagent. The Bioanalyzer (Agilent Technologies) was employed to check the quality of the total RNA. The libraries were generated using Epicentre's Ribo-Zero rRNA Removal kit to remove rRNA from total RNA. The Illumina TruSeq™ RNA Sample Prep Kit (Illumina, San Diego, CA, USA) was used to construct the library, utilising 30–100 ng of Ribo-Zero RNA. For quality analysis prior to sequencing, the generated cDNA libraries were quantified using a Qubit dsDNA HS Assay Kit and a Qubit 3.0 fluorometer (both from Thermo Fisher Scientific, Wilmington, DE, USA). The RNA libraries were subjected to sequencing as 2 × 50 bp paired-end reads with 2 samples per lane on Illumina HiSeq2500 sequencers. Reads obtained from the sequencing were aligned using STAR version 2.5.2b(1), and aligned against the human reference genome (Ensemble GRCh37). Fragments per kilobase of exon per million fragments mapped with upper quartile normalization (FPKM-UQ) were computed and adopted as the gene expression levels throughout the study.

Identification of differentially expressed genes (DEGs) and pathway analysis

The raw gene counts were determined and normalized via HTSeq(2). We set a corrected P value < 0.01 and |log2-fold change | >1 as the significance criteria to identify the DEGs by the "DESeq" package, which were displayed in volcano plots. Principal component analysis (PCA) was used to identify the differences between the two groups.

The "clusterProfiler", "org.Hs.eg.db", and "enrichplot" packages in R were used for enrichment analysis in Metascape (http://metascape.org/) to determine whether specific biological pathways were significantly overrepresented or underrepresented in the context of the experimental data(3). We compared the enrichment levels of these genes between metastatic and nonmetastatic TDLNs with the Mann–Whitney U test and set a corrected P value < 0.05 as the cutoff for significance.

Single-cell RNA sequencing sample preparation and processing

These tissues were cut into approximately 1 mm^3 pieces in RPMI-1640 medium (Invitrogen) with 10% foetal bovine serum (FBS; ScienCell) and enzymatically digested for 30 minutes on a rotor at 37°C using a MACS tumor dissociation kit (Miltenyi Biotec), according to the manufacturer's instructions. The suspended cells were centrifuged at 400 g for 5 minutes after filtration with a 70 m Cell-Strainer (BD) in RPMI-1640 media (Invitrogen). The pelleted cells were suspended in red blood cell lysis buffer (Solarbio) and incubated on ice for 2 minutes to lyse red blood cells after the supernatant was removed. After washing twice with PBS (Invitrogen), the cell pellets were resuspended in sorting buffer (PBS mixed with 2% FBS). The single cell suspensions were stained for flow cytometry (FACS) on a BD Aria III equipment with 7-AAD Viability Staining Solution (Cat# 00-6993-50, eBiocience). Live cells were sorted into 1.5 ml tubes with sorting buffer and manually counted under the microscope based on FACS analysis. The GemCode Single Cell Platform was then used to process single cells using the 3' GemCode Gel Bead, Chip, and Library Kits (10 x Genomics) according to the manufacturer's methodology. For each sample, the loaded cell numbers were 10,000. The cells were then partitioned into Emulsion Gel Beads in the GemCode instrument, where they were lysed and barcodes were ligated by reverse transcription; the RNA was then amplified and sheared, and 3' adaptors and sample indexes were ligated. The libraries were sequenced on an Illumina HiSeq 4000 using a 150bp paired-end approach.

The libraries for single-cell RNA sequencing were prepared using the Chromium Single Cell 3′ v3 according to the manufacturer’s protocol (10x Genomics). A total of 7,000 cells were targeted per sample. Libraries were sequenced on the NextSeq 500 platform (Illumina) with paired-end sequencing.

Single-cell RNA sequencing and cell type determination

The raw fast-q files were mapped to the reference genome GRCh38 using Cell Ranger Single Cell (version 5.0.1) software with the default parameters. The filtered

results were used as input for the second round of quality control and downstream analysis in Seurat (version 4.0)(4). To exclude potential cell debris and doublets from the dataset, we filtered out cells with nFeatrue_RNA ≤100 and ≥6000. Potential dead cells with high expression of mitochondrial genes (≥25%) were also eliminated from the following analysis. To reduce the potential effect on the cell clustering results, we removed genes expressed in fewer than 3 cells, as well as mitochondrial genes and ribosome-associated genes. The filtered genes were projected into principal component space using principal component analysis (RunPCA). The top 50 significant dimensions identified by ElbowPlot were subjected to graph-based clustering. Uniform manifold approximation and projection (UMAP) was utilized in the dimensionality reduction of cells for visualization, and a shared nearest neighbor-based clustering algorithm was used to find the clusters. FindAllMarkers was used to identify the cell type specific genes with the parameters logfc.threshold = 0.25, only.pos = TRUE, and min.pct = 0.25. By integrating the cell type-specific markers identified in previous studies(5, 6), validated cell markers curated in CellMarker and cell type-specific genes, we assigned a cell type identity to each cell cluster.

Cell type-specific variations in metastatic TDLNs

The FindMarker function was used to examine the cell type (clusters) changes in primary tumors and TDLNs with min.pct = 0.25, logfc.threshold = 0.25, and test.use = "wilcox". The genes with p_val_adj ≤ 0.01 were considered significantly differentially expressed. The clusterProfiler package was used to evaluate the over-representation of DEGs in each cluster.

The average gene expression levels between cell types of lymph nodes were evaluated using the AverageExpression function according to the RNA assays. And then the cell type-specific expression of the DEGs between metastatic and nonmetastatic TDLNs was estimated according to this normalized gene expression matrix. Further cell-type-specific enrichment analysis of the DEGs in B cells, CD4+ T cells, CD8+ T cells, and macrophages was performed using the Gene Ontology

(GO) and Kyoto Encyclopedia of Genes and Genomes (KEGG) databases to clarify the functional changes in each immune cell type following LNM.

To predict the cell type variation between metastatic and nonmetastatic TDLNs, CIBERSORTx(7) was used to deconvolute the bulk RNA-seq data by using our sc-RNA-seq data as a reference. Briefly, random sampling of 1,000 cells for each cell type was performed, and the related data were combined to construct the reference matrix. This read count matrix was then used to deconvolute the normalized bulk RNA-seq data via CIBERSORTx with default settings. The S-mode was selected to remove any potential batch effects.

Proteomics sequencing sample preparation and processing

BSA standard protein solution was prepared according to the instructions of Bradford protein quantitative kit, with gradient concentration ranged from 0 to 0.5 g/L. BSA standard protein solutions and sample solutions with different dilution multiples were added into 96-well plate to fill up the volume to 20 μL, respectively. Each gradient was repeated three times. The plate was added 180 μL G250 dye solution quickly and placed at room temperature for 5 minutes, the absorbance at 595 nm was detected. The standard curve was drawn with the absorbance of standard protein solution and the protein concentration of the sample was calculated. 20 μg of the protein sample was loaded to 12% SDS-PAGE gel electrophoresis, wherein the concentrated gel was performed at 80 V for 20 min, and the separation gel was performed at 120 V for 90 min. The gel was stained by coomassie brilliant blue R-250 and decolored until the bands were visualized clearly.

Each protein sample was taken and the volume was made up to 100 μL with DB lysis buffer (8 M Urea, 100 mM TEAB, pH 8.5), trypsin and 100 mM TEAB buffer were added, sample was mixed and digested at 37 °C for 4 h(8). Then trypsin and CaCl2 were added digested overnight. Formic acid was mixed with digested sample, adjusted pH under 3, and centrifuged at 12000 g for 5 min at room temperature. The supernatant was slowly loaded to the C18 desalting column, washed with washing buffer (0.1% formic acid, 3% acetonitrile) 3 times, then added elution buffer (0.1%

formic acid, 70% acetonitrile). The eluents of each sample were collected and lyophilized.

An appropriate amount of peptide was taken from each sample and separated by chromatography using Vanquish Neo UHPLC system, operated Neo UHPLC chromatography system (Thermo Scientific). Buffers: Liquid A is 0.1% formic acid in water, Liquid B is 0.1% formic acid in acetonitrile in water (80% acetonitrile).The chromatographic column was equilibrated with 96% of liquid A. Samples were injected into Trap Column (PepMap Neo 5 μm C18 300 μm X 5 mm, Thermo Scientific) and passed through a chromatography column (μPAC Neo High Throughput column, Thermo Scientific) for gradient separation. The liquid phase gradient was set as follows: 0min-0.1min, liquid B linear gradient from 4%-6%; 0.1min-1.1min, liquid B linear gradient from 6%-12%; 1.1min-4.3min, liquid B linear gradient from 12%-22.5%; 4.3min-6.1min, liquid B linear gradient from 22.5%-45%; 6.1min-8min, liquid B maintained at 99%. Peptides were separated and analyzed by DIA (data-independent acquisition) mass spectrometry using an Orbitrap Astral mass spectrometer (Thermo Scientific). Analysis time is 8 min, electrospray Voltage: 2.2kV, Detection mode: Positive Ion, Parent Ion Scanning Range: 380-980m/z, MS1 Resolution: 240000, AGC target: 500%, MS1 Maximum IT: 3 ms, MS2 Resolution: 80000, AGC target: 500%, MS2 Maximum IT: 3 ms, RF-lens: 40%, MS2 Activation Type: HCD, Isolation window: 2 Th, Normalized collision energy: 25%, cycle time: 0.6.

Proteomic sequencing

The raw DIA data were processed and analyzed by Spectronaut 17 (Biognosys AG, Switzerland) with the default settings. The Clusters of Orthologous Groups of Proteins (COG) database, GO database, and KEGG database were downloaded from UniProt/NCBI. Trypsin was used as the digestion enzyme, and the digestion type was specific. Carbamidomethyl on cysteine was specified as the fixed modification. Oxidation of methionine was specified as a variable modification. The retention time prediction type was set to dynamic iRT. Data extraction was determined by

Spectronaut based on extensive mass calibration. Spectronaut can dynamically determine the ideal extraction window depending on the iRT calibration and gradient stability. The Q value (FDR) cutoff at the precursor level was 1%, and that at the protein level was 1%. Decoy generation was set to a mutation similar to that of the scrambled sequence, but a random number of AA position swamps was used (min=2, max=length/2). The normalization strategy was set to local normalization. Peptides that passed the 1% Q value cutoff were used to calculate the major group quantities via the MaxLF Q method. We set the corrected P value < 0.05 and |log2-fold change| >1 as the significance criteria to identify the differentially expressed proteins (DEPs), which are displayed in volcano plots. PCA was used to identify the differences in tissue origins (Supplementary Figure 11).

Gene set enrichment analysis (GSEA) was used to identify enriched pathways and explore biological processes. Gene Ontology (GO) functional enrichment analysis was conducted using the InterProScan program with nonredundant protein data as a reference (data were from sources including Pfam, PRINTS, ProDom, SMART, ProSite, PANTHER)(9), and Kyoto Encyclopedia of Genes and Genomes (KEGG) pathway enrichment analysis was performed. The DEPs were subjected to volcano map analysis, cluster heatmap analysis and GO and KEGG enrichment analysis(10).

H&E and IHC staining sample preparation and processing

All the tissues were formalin-fixed and embedded in paraffin to create 4 μm paraffin sections. These sections underwent processing steps, including deparaffinization, rehydration and endogenous peroxidase inactivation, followed by antigen retrieval using sodium citrate buffer (10 mM, pH 6.0) with microwave treatment at 95 °C for 20 min.

For H&E staining, tissue sections were incubated in acidic hemalum staining solution (Waldeck) for 8 minutes, washed and incubated in eosin staining solution (Sigma-Aldrich) for 2.5 minutes at room temperature using a Tissue-Tek Prisma Plus slide stainer (Sakura).

For immunohistochemistry (IHC), CD3 antibodies were purchased from ZSGS-Bio (dilution 1:800, ZA0503, Beijing, China), CD8 antibodies from Monosan (dilution 1:300, C8/144B, The Kingdom of the Netherlands), and CD19 antibodies from Cell Signaling Technology (dilution 1:800, # 90176 S, Massachusetts, USA). Positive controls, human tonsil tissues, were stained with these antibodies, while isotype-matched antibodies were used as negative controls. The IHC staining procedure was automated using the BenchMark ULTRA immunostainer from Ventana Medical Systems, Inc. The CD3, CD8, and CD19 expression was evaluated in both the IF and CT of primary tumor and live metastases, as well as TDLNs.

MSI status evaluation

The Microsatellite instability (MSI) status of 428 patients was evaluated independently using IHC. Four mismatch repair (MMR) proteins (MLH1, MSH2, MSH6, and PMS2) were stained in tumor samples, and the results were evaluated independently by two pathologists. The patients who showed positive staining of the nuclei of all four MMR proteins were considered as proficient MMR (pMMR)/ microsatellite stable (MSS), and the mismatch repair-deficient (dMMR) /microsatellite instability-high (MSI-H) cases show the loss of one of the two MLH1/PMS2 or MSH2/MSH6 heterodimers(11). The survival comparisons between pMMR/dMMR CRLM patients with and without LNM were performed based on the Kaplan–Meier method.

1. Holm JB, Humphrys MS, Robinson CK, Settles ML, Ott S, Fu L, et al. Ultrahigh-Throughput Multiplexing and Sequencing of >500-Base-Pair Amplicon Regions on the Illumina HiSeq 2500 Platform. mSystems (7324). 2019;4(1).
2. Bao Y, Wang L, Shi L, Yun F, Liu X, Chen Y, et al. Transcriptome profiling revealed multiple genes and ECM-receptor interaction pathways that may be associated with breast cancer. Cell Mol Biol Lett. 2019;24:38.
3. Hänzelmann S, Castelo R, Guinney J. GSVA: gene set variation analysis for microarray and RNA-seq data. BMC BIOINFORMATICS (3307). 2013;14:7.
4. Hao Y, Stuart T, Kowalski MH, Choudhary S, Hoffman P, Hartman A, et al. Dictionary learning for integrative, multimodal and scalable single-cell analysis. Nat Biotechnol. 2024;42(2):293-304.
5. Che LH, Liu JW, Huo JP, Luo R, Xu RM, He C, et al. A single-cell atlas of liver metastases of

colorectal cancer reveals reprogramming of the tumor microenvironment in response to preoperative chemotherapy. Cell Discov. 2021;7(1):80.
6. Pelka K, Hofree M, Chen JH, Sarkizova S, Pirl JD, Jorgji V, et al. Spatially organized multicellular immune hubs in human colorectal cancer. CELL (66850). 2021;184(18):4734-52.e20.
7. Newman AM, Liu CL, Green MR, Gentles AJ, Feng W, Xu Y, et al. Robust enumeration of cell subsets from tissue expression profiles. Nat Methods. 2015;12(5):453-7.
8. Zhang H, Liu T, Zhang Z, Payne SH, Zhang B, McDermott JE, et al. Integrated Proteogenomic Characterization of Human High-Grade Serous Ovarian Cancer. CELL (66850). 2016;166(3):755-65.
9. Jones P, Binns D, Chang HY, Fraser M, Li W, McAnulla C, et al. InterProScan 5: genome-scale protein function classification. BIOINFORMATICS (6931). 2014;30(9):1236-40.
10. Huang da W, Sherman BT, Lempicki RA. Bioinformatics enrichment tools: paths toward the comprehensive functional analysis of large gene lists. Nucleic Acids Res. 2009;37(1):1-13.
11. Samowitz WS. Evaluation of colorectal cancers for Lynch syndrome: practical molecular diagnostics for surgical pathologists. Mod Pathol. 2015;28 Suppl 1:S109-13.

Supplementary Figure 1 Protocol for Halo image analysis.

(A) Halo image analysis automatically identifies IHC-positive cells in the TDLNs. (B) Pearson correlation analysis between manual counting and AI counting, showing the accuracy of Halo image analysis. (C) Halo image analysis automatically identifies IHC-positive cells at the IF of liver metastasis. (D) Pearson correlation analysis between manual counting and AI counting, showing the accuracy of Halo image analysis.

Abbreviations: TDLNs, tumor-draining lymph nodes; AI, artificial intelligence; IHC, immunohistochemistry; IF, invasive front.

Supplementary Figure 2 Transcriptome differences in TDLNs.

(A) Volcano plot showing DEGs in TDLNs; (B) Pathways enriched in upregulated DEGs in positive TDLNs based on the Metascape enrichment method. (C) Pathways enriched in downregulated DEGs in positive TDLNs based on the Metascape enrichment method.

Abbreviations: TDLNs, tumor-draining lymph nodes; DEGs, differentially expressed genes.

Supplementary Figure 3 Transcriptomic characterization of TDLNs.

(A) Uniform manifold approximation and projection (UMAP) analysis of transcriptional profiles, colored by cell type and stratified by sample type. (B) Violin plots showing marker gene expression of each cell subtype. (C) Box plots illustrating

the differences in nonimmune cell infiltration between NegLNs and PosLNs using lymph node sc-RNA sequencing data as a reference based on CIBERSORTx; (D) Heatmap and box plots showing highly expressed DEGs and downregulated DEGs (PosLN vs. NegLN) across cell types.

Abbreviations: NegLN, negative lymph node; PosLN, positive lymph node; sc-RNA, single-cell RNA; DEGs, differentially expressed genes.

Supplementary Figure 4 Cell type-specific DEGs and enrichment analysis in TDLNs.

(A) Dot plot showing cell type-specific DEGs in TDLNs; (B) Pathways enriched in downregulated B Cell-specific DEGs in positive TDLNs; (C) Pathways enriched in downregulated CD4+ T Cell-specific DEGs in positive TDLNs; (D) Pathways enriched in downregulated CD8+ T Cell-specific DEGs in positive TDLNs; (E) Pathways enriched in downregulated Macrophage-specific DEGs in positive TDLNs.

Abbreviations: DEGs, differentially expressed genes; TDLNs, tumor-draining lymph nodes;

Supplementary Figure 5 Cell type-specific enrichment analysis in TDLNs.

(A) Pathways enriched in upregulated B Cell-specific DEGs in positive TDLNs; (B) Pathways enriched in upregulated CD4+ T Cell-specific DEGs in positive TDLNs; (C) Pathways enriched in upregulated CD8+ T Cell-specific DEGs in positive TDLNs; (D) Pathways enriched in upregulated Macrophage-specific DEGs in positive TDLNs.

Abbreviations: DEGs, differentially expressed genes; TDLNs, tumor-draining lymph nodes;

Supplementary Figure 6 Proteomic characteristics of positive and negative TDLNs.

(A) Violin plots verifying protein differences in nonimmune cell-associated upregulated genes. (B-C) GSEA plot of the (B) Th1 and Th2 cell differentiation gene sets and (C) Th17 cell differentiation gene set. (D) Violin plot showing the difference in protein expression between PosLNs and NegLNs in these two pathway gene sets. (E) The dot plot shows the KEGG pathways enriched in the proteins downregulated in NegLNs. (F) GSEA plot of the ECM receptor interaction gene set. (G) Violin plot showing the difference in protein expression between PosLNs and NegLNs in the ECM receptor interaction gene set.

Abbreviations: NegLN, negative lymph node; PosLN, positive lymph node; Th, helper T cell; ECM, extracellular matrix; TDLNs, tumor-draining lymph nodes; GSEA, gene set enrichment analysis; KEGG, Kyoto Encyclopedia of Genes and Genomes.

Supplementary Figure 7 Morphological features of TDLNs.

(A) Differences in the morphology of negative and positive TDLNs: (B) pathological images, (F) bar plot of the quantitative results.

Abbreviations: NegLN, negative lymph node; PosLN, positive lymph node; LNM,

lymph node metastasis; GC, germinal center; TDLNs, tumor-draining lymph nodes.

Supplementary Figure 8 Proteomic and pathological characteristics of primary tumors with and without LNM.

(A) Heatmap of differentially expressed proteins in primary tumors with and without LNM. (B) Volcano plot showing differentially expressed proteins in primary tumors with and without LNM. (C-D) Bar plot illustrating the GO terms enriched in the differentially expressed proteins in primary tumors: (C) downregulated DEGs, (D) upregulated DEGs. (E) Violin plot showing the quantitative results of IHC staining in the center. (F-G) Violin plot showing the quantitative results of CLR: (F) the number of CLRs and (G) the length of the IF. (H) The bar plot shows the dMMR status of the primary tumor with and without LNM.

Abbreviations: GO, gene ontology; BP, biological process; CC, cellular component; MF, molecular function; LNM, lymph node metastasis; CLR, Crohn-like lymphoid reaction; IF, invasive front; pMMR, proficient mismatch repair; dMMR, deficient mismatch repair; IHC, immunohistochemistry.

Supplementary Figure 9 Survival comparisons between pMMR/dMMR CRLM patients with and without LNM.

(A) Kaplan–Meier curve of stratified survival in pMMR CRLM patients; (B) Kaplan–Meier curve of stratified survival in dMMR CRLM patients.

Abbreviations: pMMR, proficient mismatch repair; dMMR, deficient mismatch

repair; CRLM, colorectal cancer liver metastasis; LNM, lymph node metastasis.

Supplementary Figure 10 Proteomic and pathological characteristics of liver metastasis with and without LNM.

(A) Bar plot illustrating the GO terms enriched in the proteins upregulated in liver metastasis with LNM. (B-C) Violin plot showing the quantitative results of IHC staining (B) in the center, (C) at the IF.

Abbreviations: GO, Gene Ontology; BP, biological process; CC, cellular component; MF, molecular function; LNM, lymph node metastasis; IF, invasive front; IHC, immunohistochemistry.

Supplementary Figure 11 Proteomic data.

(A) Sample correlation heatmap showing tissue heterogeneity between samples. (B) PCA dimensionality reduction plot of samples using proteomic sequencing as a feature. (C) Proteomics data annotation results based on the KEGG database. (D-E) Bar plot illustrating the GO terms enriched in the proteins: (D) downregulated proteins and (E) upregulated proteins.

Abbreviations: LNM, lymph node metastasis; GO, Gene Ontology; PCA, principal component analysis; KEGG, Kyoto Encyclopedia of Genes and Genomes.

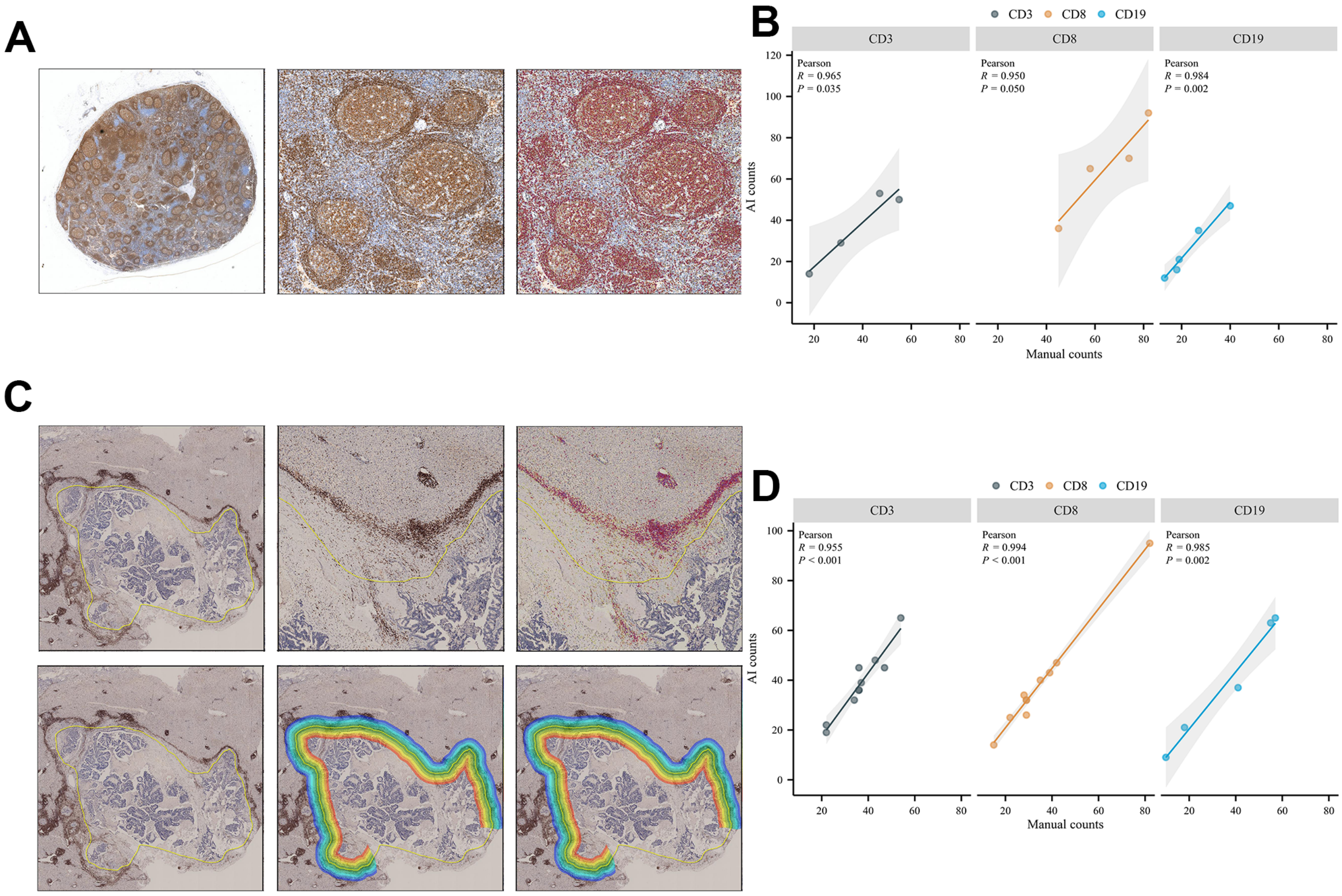
A
B
CD3
CD8
CD19
Pearson
R = 0.965
P = 0.035
Pearson
R = 0.950
P = 0.050
Pearson
R = 0.984
P = 0.002
AI counts
Manual counts
0
20
40
60
80
100
120
C
D
Pearson
R = 0.955
P < 0.001
Pearson
R = 0.994
P < 0.001
Pearson
R = 0.985
P = 0.002

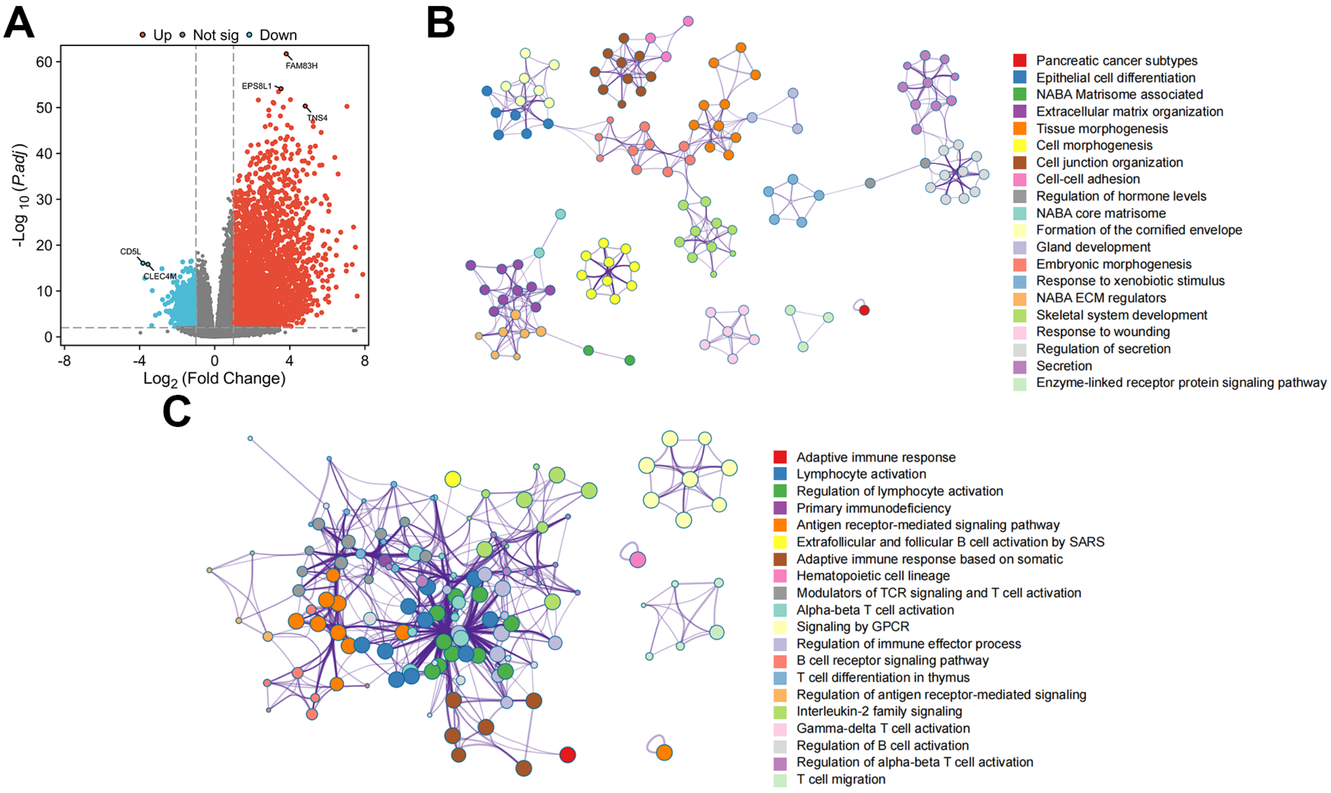
A
Up
Not sig
Down
60
50
40
30
20
10
0
-Log 10 (P.adj)
-8
-4
0
4
8
Log2 (Fold Change)
FAM83H
EPS8L1
TNS4
CD5L
CLEC4M
B
Pancreatic cancer subtypes
Epithelial cell differentiation
NABA Matrisome associated
Extracellular matrix organization
Tissue morphogenesis
Cell morphogenesis
Cell junction organization
Cell-cell adhesion
Regulation of hormone levels
NABA core matrisome
Formation of the cornified envelope
Gland development
Embryonic morphogenesis
Response to xenobiotic stimulus
NABA ECM regulators
Skeletal system development
Response to wounding
Regulation of secretion
Secretion
Enzyme-linked receptor protein signaling pathway
C
Adaptive immune response
Lymphocyte activation
Regulation of lymphocyte activation
Primary immunodeficiency
Antigen receptor-mediated signaling pathway
Extrafollicular and follicular B cell activation by SARS
Adaptive immune response based on somatic
Hematopoietic cell lineage
Modulators of TCR signaling and T cell activation
Alpha-beta T cell activation
Signaling by GPCR
Regulation of immune effector process
B cell receptor signaling pathway
T cell differentiation in thymus
Regulation of antigen receptor-mediated signaling
Interleukin-2 family signaling
Gamma-delta T cell activation
Regulation of B cell activation
Regulation of alpha-beta T cell activation
T cell migration

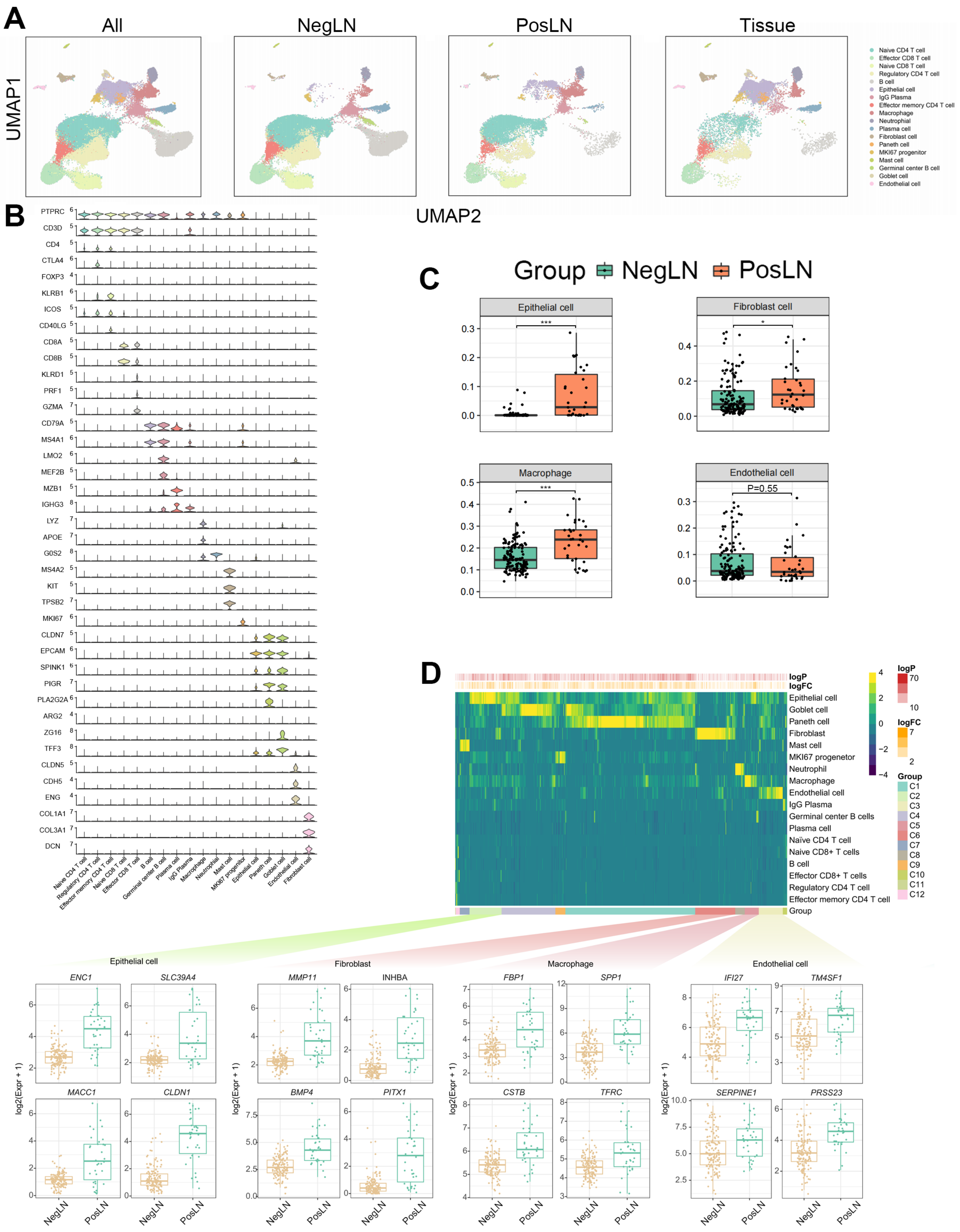

A
All
NegLN
PosLN
Tissue
UMAP1
UMAP2
Naive CD4 T cell
Effector CD8 T cell
Naive CD8 T cell
Regulatory CD4 T cell
B cell
Epithelial cell
IgG Plasma
Effector memory CD4 T cell
Macrophage
Neutrophial
Plasma cell
Fibroblast cell
Paneth cell
MKI67 progenitor
Mast cell
Germinal center B cell
Goblet cell
Endothelial cell
B
PTPRC
CD3D
CD4
CTLA4
FOXP3
KLRB1
ICOS
CD40LG
CD8A
CD8B
KLRD1
PRF1
GZMA
CD79A
MS4A1
LMO2
MEF2B
MZB1
IGHG3
LYZ
APOE
G0S2
MS4A2
KIT
TPSB2
MKI67
CLDN7
EPCAM
SPINK1
PIGR
PLA2G2A
ARG2
ZG16
TFF3
CLDN5
CDH5
ENG
COL1A1
COL3A1
DCN
C
Group NegLN PosLN
Epithelial cell
Fibroblast cell
Macrophage
Endothelial cell
P=0.55
D
logP
logFC
Epithelial cell
Goblet cell
Paneth cell
Fibroblast
Mast cell
MKI67 progenitor
Neutrophil
Macrophage
Endothelial cell
IgG Plasma
Germinal center B cells
Plasma cell
Naive CD4 T cell
Naive CD8+ T cells
B cell
Effector CD8+ T cells
Regulatory CD4 T cell
Effector memory CD4 T cell
Group
C1
C2
C3
C4
C5
C6
C7
C8
C9
C10
C11
C12
Epithelial cell
Fibroblast
Macrophage
Endothelial cell
ENC1
SLC39A4
MACC1
CLDN1
MMP11
INHBA
BMP4
PITX1
FBP1
SPP1
CSTB
TFRC
IFI27
TM4SF1
SERPINE1
PRSS23
log2(Expr + 1)
NegLN
PosLN

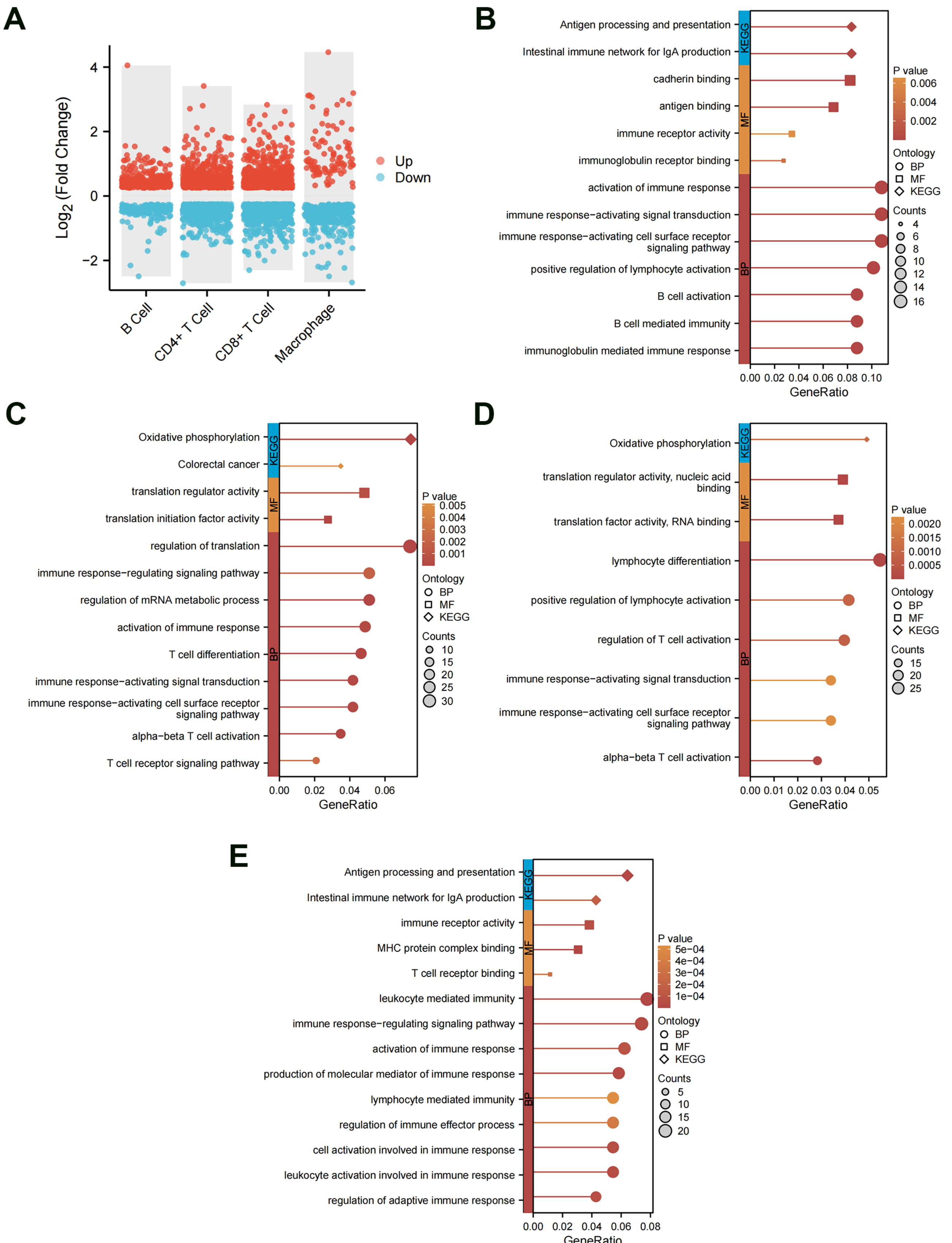
A
Log2 (Fold Change)
Up
Down
B Cell
CD4+ T Cell
CD8+ T Cell
Macrophage
B
Antigen processing and presentation
Intestinal immune network for IgA production
cadherin binding
antigen binding
immune receptor activity
immunoglobulin receptor binding
activation of immune response
immune response−activating signal transduction
immune response−activating cell surface receptor signaling pathway
positive regulation of lymphocyte activation
B cell activation
B cell mediated immunity
immunoglobulin mediated immune response
GeneRatio
P value
Ontology
BP
MF
KEGG
Counts
C
Oxidative phosphorylation
Colorectal cancer
translation regulator activity
translation initiation factor activity
regulation of translation
immune response−regulating signaling pathway
regulation of mRNA metabolic process
activation of immune response
T cell differentiation
immune response−activating signal transduction
immune response−activating cell surface receptor signaling pathway
alpha−beta T cell activation
T cell receptor signaling pathway
D
Oxidative phosphorylation
translation regulator activity, nucleic acid binding
translation factor activity, RNA binding
lymphocyte differentiation
positive regulation of lymphocyte activation
regulation of T cell activation
immune response−activating signal transduction
immune response−activating cell surface receptor signaling pathway
alpha−beta T cell activation
E
Antigen processing and presentation
Intestinal immune network for IgA production
immune receptor activity
MHC protein complex binding
T cell receptor binding
leukocyte mediated immunity
immune response−regulating signaling pathway
activation of immune response
production of molecular mediator of immune response
lymphocyte mediated immunity
regulation of immune effector process
cell activation involved in immune response
leukocyte activation involved in immune response
regulation of adaptive immune response

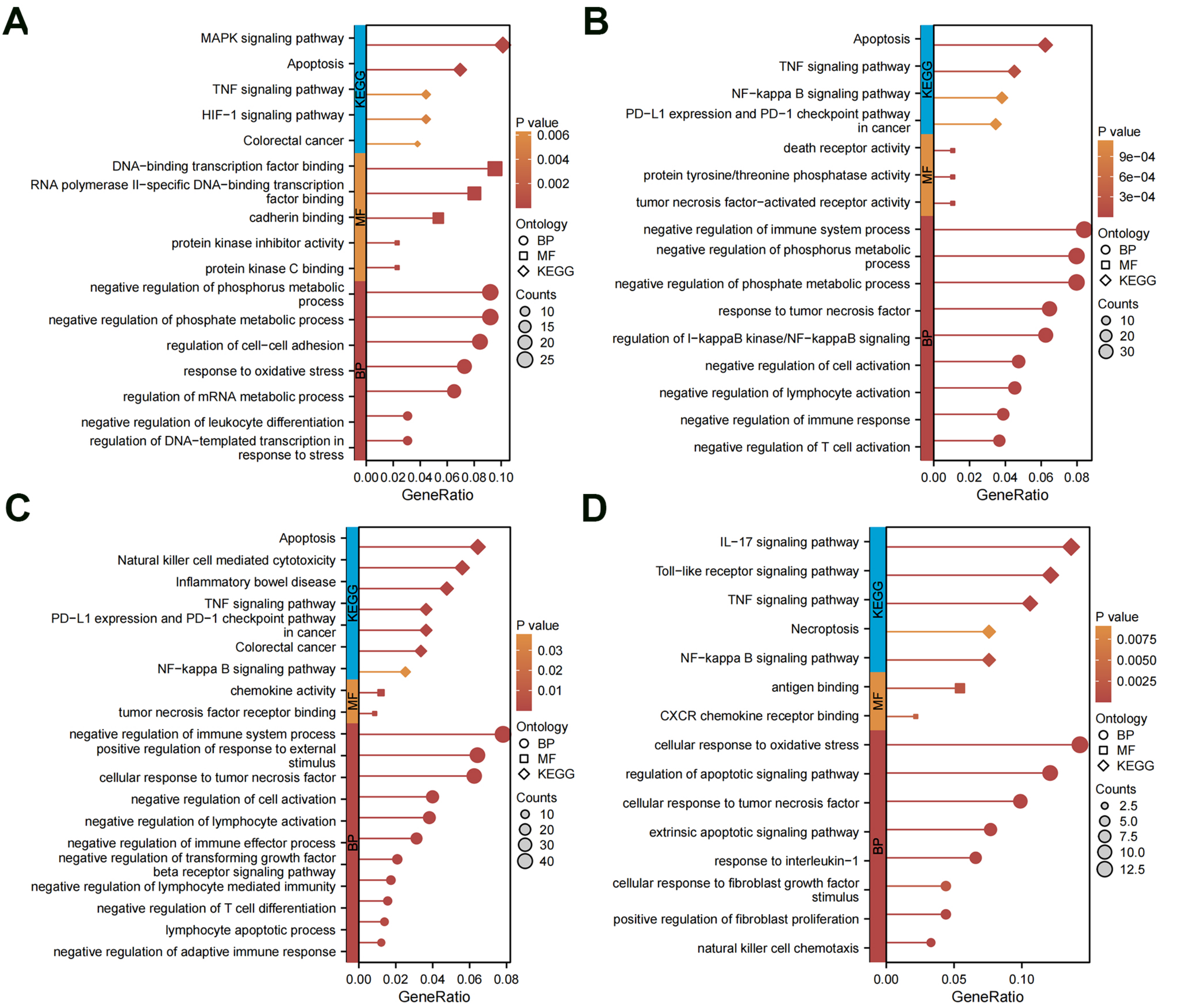
A
MAPK signaling pathway
Apoptosis
TNF signaling pathway
HIF−1 signaling pathway
Colorectal cancer
DNA−binding transcription factor binding
RNA polymerase II−specific DNA−binding transcription factor binding
cadherin binding
protein kinase inhibitor activity
protein kinase C binding
negative regulation of phosphorus metabolic process
negative regulation of phosphate metabolic process
regulation of cell−cell adhesion
response to oxidative stress
regulation of mRNA metabolic process
negative regulation of leukocyte differentiation
regulation of DNA−templated transcription in response to stress
KEGG
MF
BP
0.00 0.02 0.04 0.06 0.08 0.10
GeneRatio
P value
0.006
0.004
0.002
Ontology
BP
MF
KEGG
Counts
10
15
20
25
B
Apoptosis
TNF signaling pathway
NF−kappa B signaling pathway
PD−L1 expression and PD−1 checkpoint pathway in cancer
death receptor activity
protein tyrosine/threonine phosphatase activity
tumor necrosis factor−activated receptor activity
negative regulation of immune system process
negative regulation of phosphorus metabolic process
negative regulation of phosphate metabolic process
response to tumor necrosis factor
regulation of I−kappaB kinase/NF−kappaB signaling
negative regulation of cell activation
negative regulation of lymphocyte activation
negative regulation of immune response
negative regulation of T cell activation
KEGG
MF
BP
0.00 0.02 0.04 0.06 0.08
GeneRatio
P value
9e−04
6e−04
3e−04
Ontology
BP
MF
KEGG
Counts
10
20
30
C
Apoptosis
Natural killer cell mediated cytotoxicity
Inflammatory bowel disease
TNF signaling pathway
PD−L1 expression and PD−1 checkpoint pathway in cancer
Colorectal cancer
NF−kappa B signaling pathway
chemokine activity
tumor necrosis factor receptor binding
negative regulation of immune system process
positive regulation of response to external stimulus
cellular response to tumor necrosis factor
negative regulation of cell activation
negative regulation of lymphocyte activation
negative regulation of immune effector process
negative regulation of transforming growth factor beta receptor signaling pathway
negative regulation of lymphocyte mediated immunity
negative regulation of T cell differentiation
lymphocyte apoptotic process
negative regulation of adaptive immune response
KEGG
MF
BP
0.00 0.02 0.04 0.06 0.08
GeneRatio
P value
0.03
0.02
0.01
Ontology
BP
MF
KEGG
Counts
10
20
30
40
D
IL−17 signaling pathway
Toll−like receptor signaling pathway
TNF signaling pathway
Necroptosis
NF−kappa B signaling pathway
antigen binding
CXCR chemokine receptor binding
cellular response to oxidative stress
regulation of apoptotic signaling pathway
cellular response to tumor necrosis factor
extrinsic apoptotic signaling pathway
response to interleukin−1
cellular response to fibroblast growth factor stimulus
positive regulation of fibroblast proliferation
natural killer cell chemotaxis
KEGG
MF
BP
0.00 0.05 0.10
GeneRatio
P value
0.0075
0.0050
0.0025
Ontology
BP
MF
KEGG
Counts
2.5
5.0
7.5
10.0
12.5

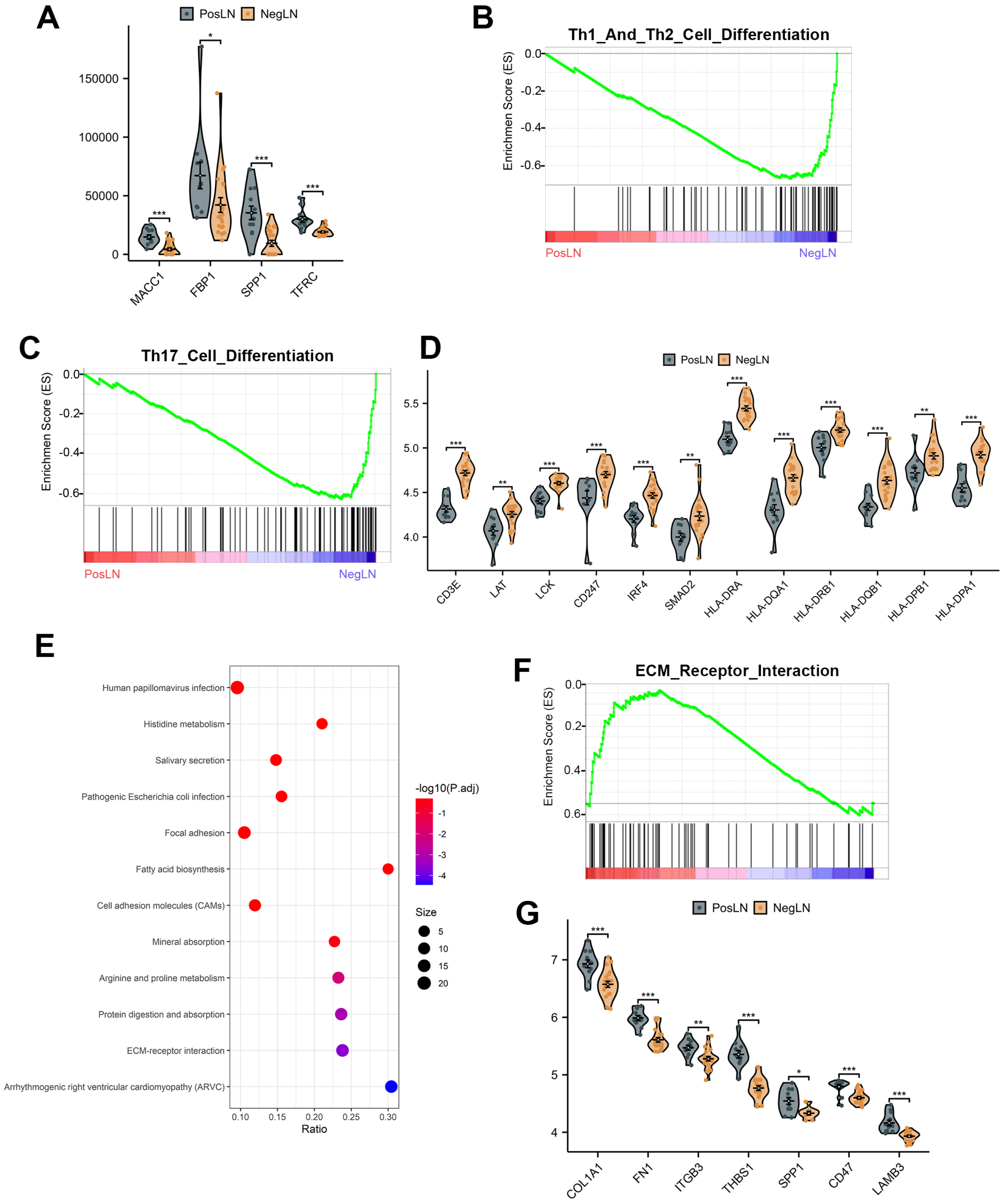

A
PosLN NegLN
150000
100000
50000
0
MACC1
FBP1
SPP1
TFRC
B
Th1_And_Th2_Cell_Differentiation
Enrichmen Score (ES)
PosLN
NegLN
C
Th17_Cell_Differentiation
Enrichmen Score (ES)
PosLN
NegLN
D
PosLN NegLN
CD3E
LAT
LCK
CD247
IRF4
SMAD2
HLA-DRA
HLA-DQA1
HLA-DRB1
HLA-DQB1
HLA-DPB1
HLA-DPA1
E
Human papillomavirus infection
Histidine metabolism
Salivary secretion
Pathogenic Escherichia coli infection
Focal adhesion
Fatty acid biosynthesis
Cell adhesion molecules (CAMs)
Mineral absorption
Arginine and proline metabolism
Protein digestion and absorption
ECM-receptor interaction
Arrhythmogenic right ventricular cardiomyopathy (ARVC)
-log10(P.adj)
Size
Ratio
F
ECM_Receptor_Interaction
Enrichmen Score (ES)
G
PosLN NegLN
COL1A1
FN1
ITGB3
THBS1
SPP1
CD47
LAMB3

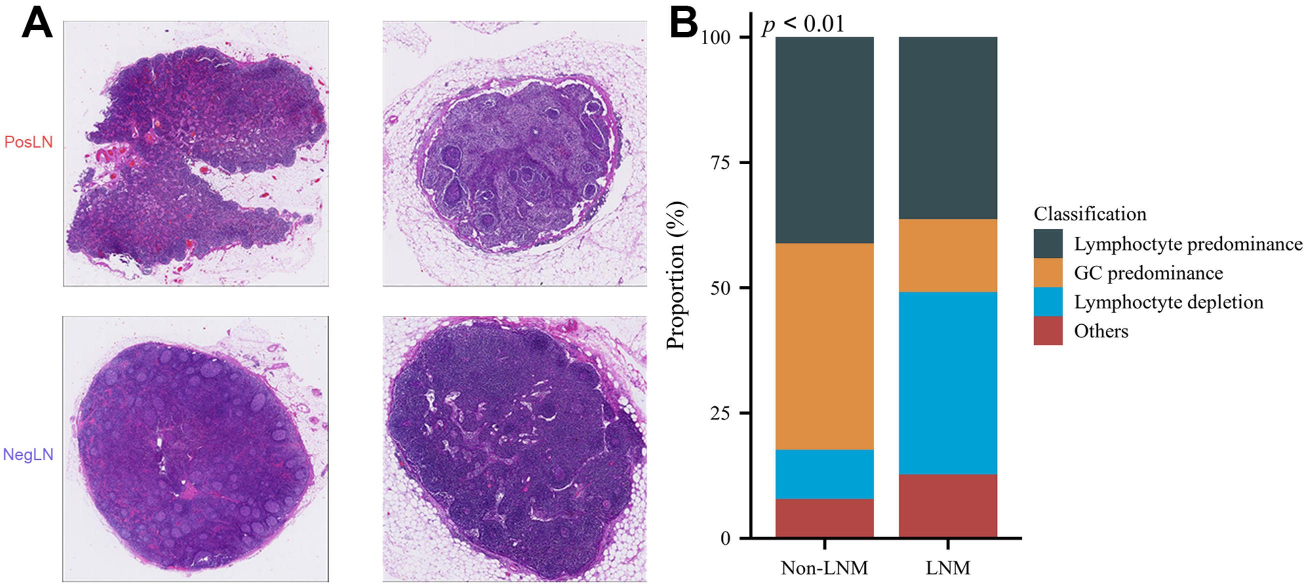
A
PosLN
NegLN
B
p < 0.01
100
75
50
25
0
Proportion (%)
Non-LNM
LNM
Classification
Lymphoctyte predominance
GC predominance
Lymphoctyte depletion
Others

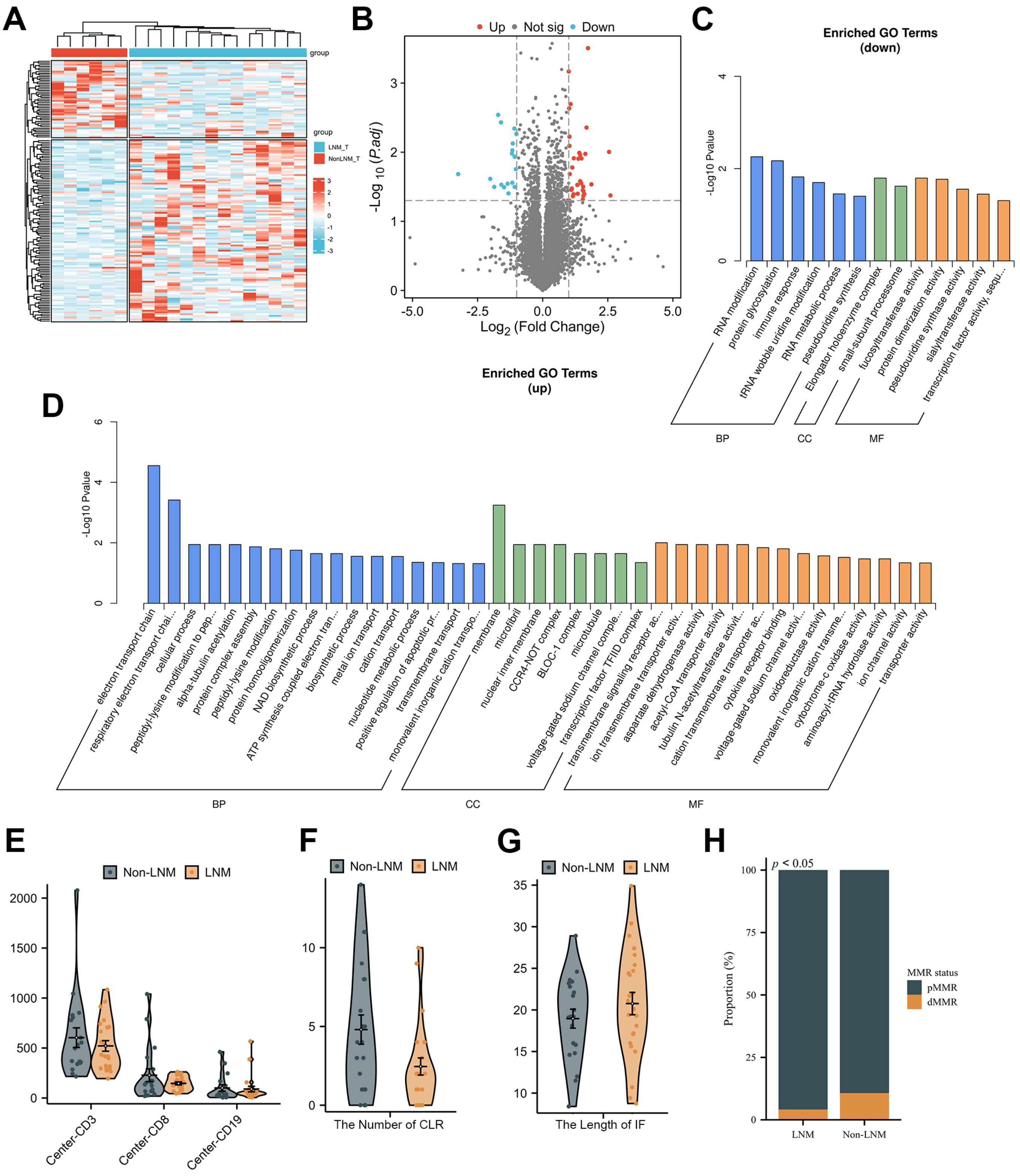

A
group
LNM_T
NonLNM_T
B
Up
Not sig
Down
-Log 10 (P.adj)
Log2 (Fold Change)
C
Enriched GO Terms (down)
-Log10 Pvalue
RNA modification
protein glycosylation
immune response
tRNA wobble uridine modification
RNA metabolic process
pseudouridine synthesis
Elongator holoenzyme complex
small-subunit processome
fucosyltransferase activity
protein dimerization activity
pseudouridine synthase activity
sialyltransferase activity
transcription factor activity, sequ...
BP
CC
MF
D
Enriched GO Terms (up)
-Log10 Pvalue
electron transport chain
respiratory electron transport chai...
cellular process
peptidyl-lysine modification to pep...
alpha-tubulin acetylation
protein complex assembly
peptidyl-lysine modification
protein homooligomerization
NAD biosynthetic process
ATP synthesis coupled electron tran...
biosynthetic process
metal ion transport
cation transport
nucleotide metabolic process
positive regulation of apoptotic pr...
transmembrane transport
monovalent inorganic cation transpo...
membrane
microfibril
nuclear inner membrane
CCR4-NOT complex
BLOC-1 complex
microtubule
voltage-gated sodium channel comple...
transcription factor TFIID complex
transmembrane signaling receptor ac...
ion transmembrane transporter activ...
aspartate dehydrogenase activity
acetyl-CoA transporter activity
tubulin N-acetyltransferase activit...
cation transmembrane transporter ac...
cytokine receptor binding
voltage-gated sodium channel activi...
oxidoreductase activity
monovalent inorganic cation transme...
cytochrome-c oxidase activity
aminoacyl-tRNA hydrolase activity
ion channel activity
transporter activity
BP
CC
MF
E
Non-LNM
LNM
Center-CD3
Center-CD8
Center-CD19
F
Non-LNM
LNM
The Number of CLR
G
Non-LNM
LNM
The Length of IF
H
p < 0.05
Proportion (%)
MMR status
pMMR
dMMR
LNM
Non-LNM

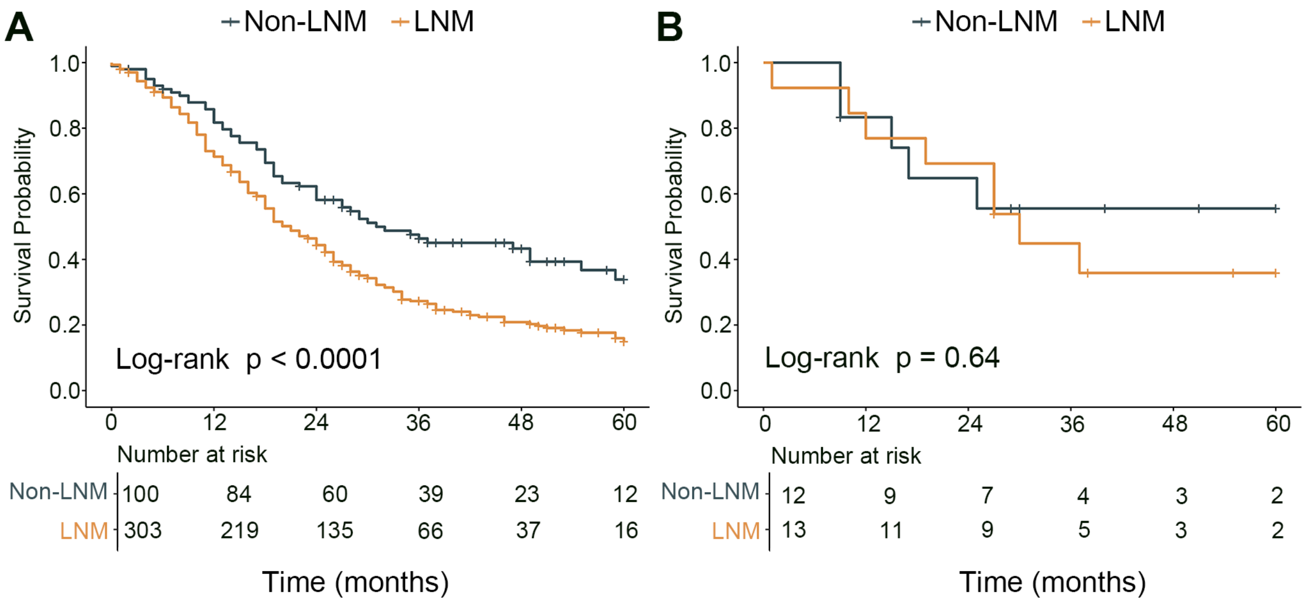
A
Non-LNM
LNM
Survival Probability
1.0
0.8
0.6
0.4
0.2
0.0
Log-rank p < 0.0001
0
12
24
36
48
60
Number at risk
Non-LNM 100 84 60 39 23 12
LNM 303 219 135 66 37 16
Time (months)
B
Non-LNM
LNM
Survival Probability
1.0
0.8
0.6
0.4
0.2
0.0
Log-rank p = 0.64
0
12
24
36
48
60
Number at risk
Non-LNM 12 9 7 4 3 2
LNM 13 11 9 5 3 2
Time (months)

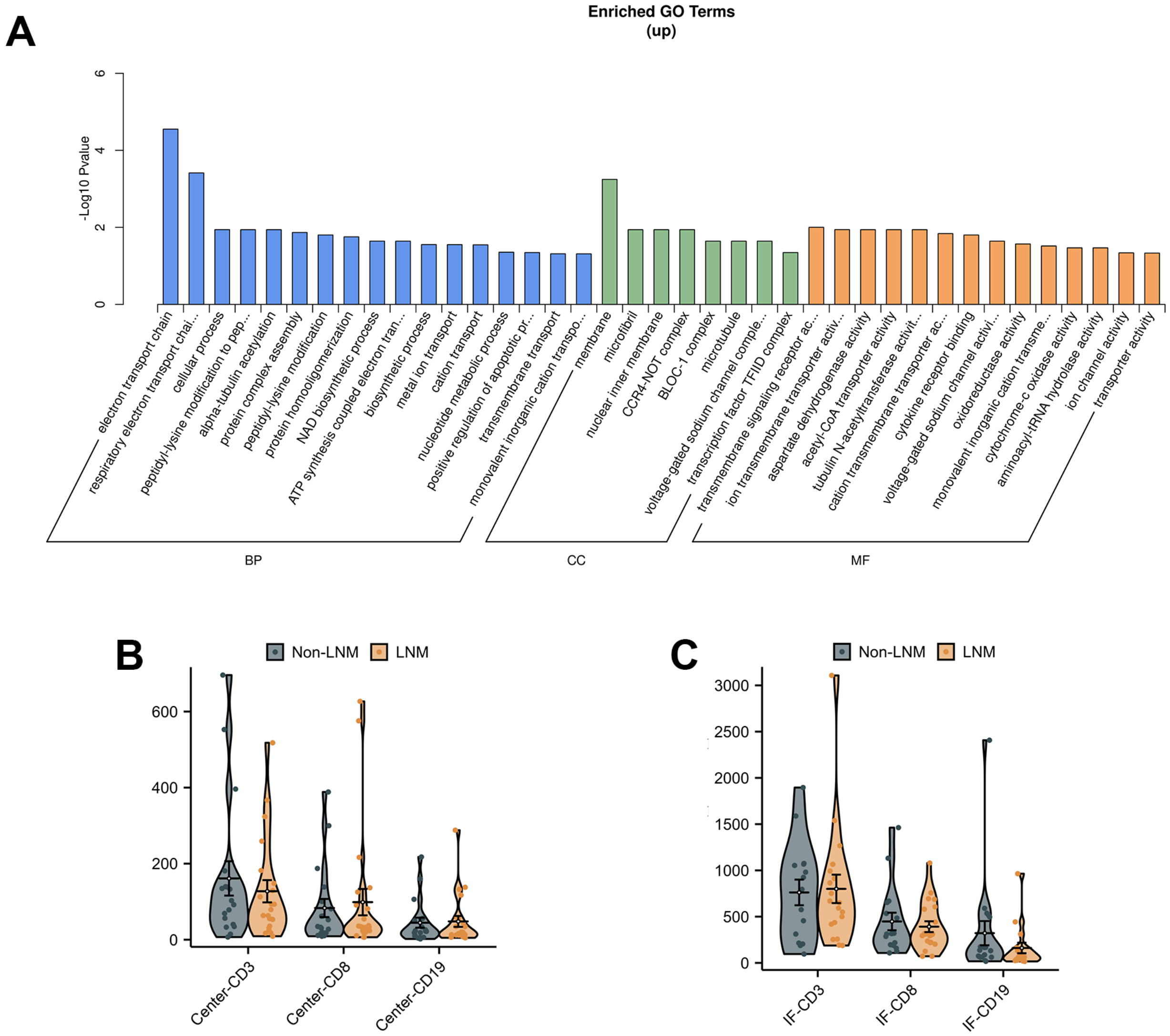
A
Enriched GO Terms
(up)
-Log10 Pvalue
0
2
4
6
electron transport chain
respiratory electron transport chai...
cellular process
peptidyl-lysine modification to pep...
alpha-tubulin acetylation
protein complex assembly
peptidyl-lysine modification
protein homooligomerization
NAD biosynthetic process
ATP synthesis coupled electron tran...
biosynthetic process
metal ion transport
cation transport
nucleotide metabolic process
positive regulation of apoptotic pr...
transmembrane transport
monovalent inorganic cation transpo...
membrane
microfibril
nuclear inner membrane
CCR4-NOT complex
BLOC-1 complex
microtubule
voltage-gated sodium channel comple...
transcription factor TFIID complex
transmembrane signaling receptor ac...
ion transmembrane transporter activ...
aspartate dehydrogenase activity
acetyl-CoA transporter activity
tubulin N-acetyltransferase activit...
cation transmembrane transporter ac...
cytokine receptor binding
voltage-gated sodium channel activi...
oxidoreductase activity
monovalent inorganic cation transme...
cytochrome-c oxidase activity
aminoacyl-tRNA hydrolase activity
ion channel activity
transporter activity
BP
CC
MF
B
Non-LNM
LNM
0
200
400
600
Center-CD3
Center-CD8
Center-CD19
C
Non-LNM
LNM
0
500
1000
1500
2000
2500
3000
IF-CD3
IF-CD8
IF-CD19

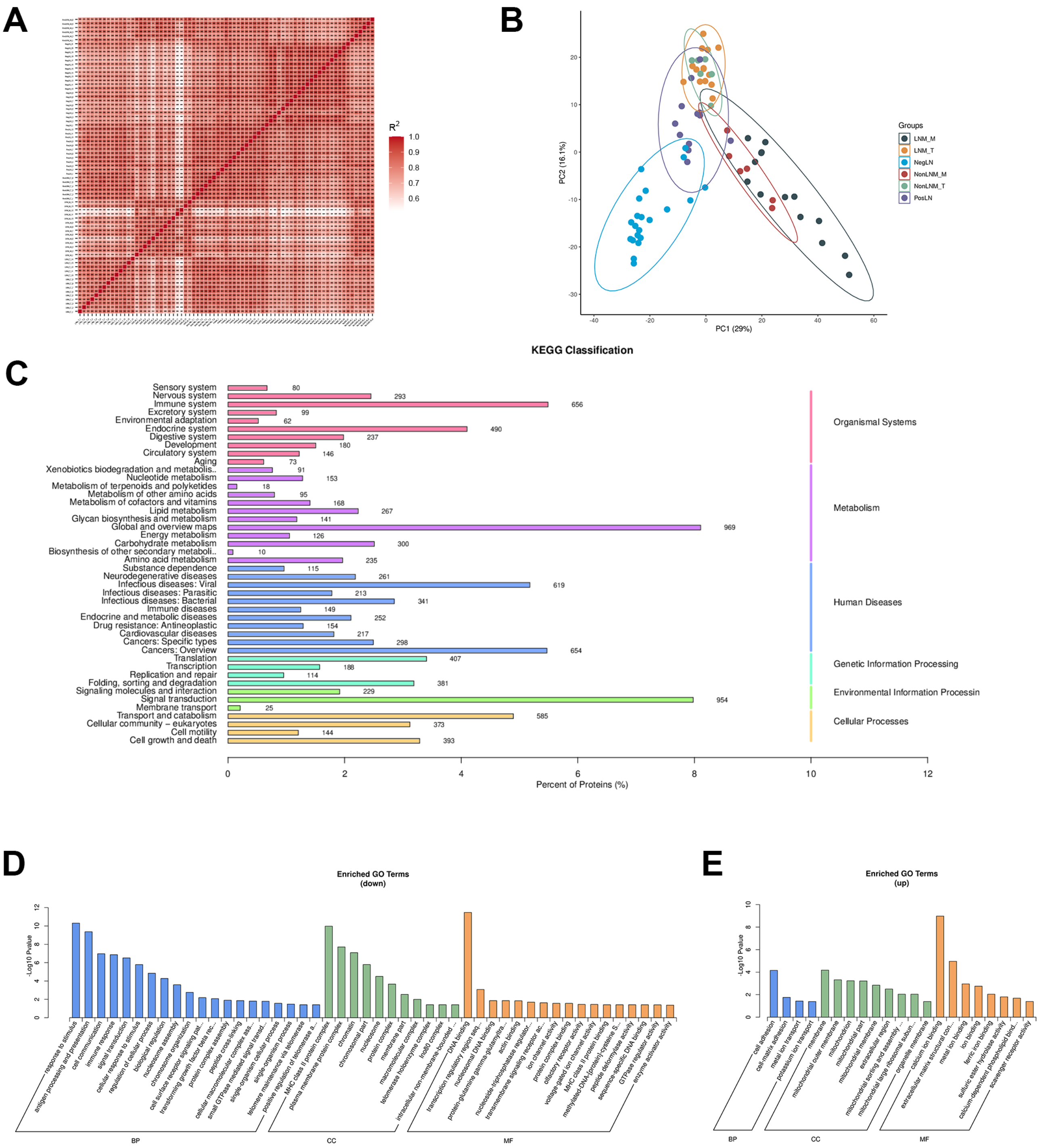
A
R2
1.0
0.9
0.8
0.7
0.6
B
PC2 (16.1%)
PC1 (29%)
Groups
LNM_M
LNM_T
NegLN
NonLNM_M
NonLNM_T
PosLN
KEGG Classification
C
Sensory system 80
Nervous system 293
Immune system 656
Excretory system 99
Environmental adaptation 62
Endocrine system 490
Digestive system 237
Development 180
Circulatory system 146
Aging 73
Xenobiotics biodegradation and metabolis.. 91
Nucleotide metabolism 153
Metabolism of terpenoids and polyketides 18
Metabolism of other amino acids 95
Metabolism of cofactors and vitamins 168
Lipid metabolism 267
Glycan biosynthesis and metabolism 141
Global and overview maps 969
Energy metabolism 126
Carbohydrate metabolism 300
Biosynthesis of other secondary metaboli.. 10
Amino acid metabolism 235
Substance dependence 115
Neurodegenerative diseases 261
Infectious diseases: Viral 619
Infectious diseases: Parasitic 213
Infectious diseases: Bacterial 341
Immune diseases 149
Endocrine and metabolic diseases 252
Drug resistance: Antineoplastic 154
Cardiovascular diseases 217
Cancers: Specific types 298
Cancers: Overview 654
Translation 407
Transcription 188
Replication and repair 114
Folding, sorting and degradation 381
Signaling molecules and interaction 229
Signal transduction 954
Membrane transport 25
Transport and catabolism 585
Cellular community – eukaryotes 373
Cell motility 144
Cell growth and death 393
Organismal Systems
Metabolism
Human Diseases
Genetic Information Processing
Environmental Information Processin
Cellular Processes
Percent of Proteins (%)
D
Enriched GO Terms
(down)
-Log10 Pvalue
BP
CC
MF
E
Enriched GO Terms
(up)
-Log10 Pvalue
BP
CC
MF

Supplementary Table 1 Baseline characteristics.

Characteristics	SEER Database<br>No. (%)	Chinese Registry<br>No. (%)
**Total**	8035	1305
**LNM**		
Yes	6883 (85.7)	974 (74.6)
No	1152 (14.3)	331 (25.4)
**Age, years**		
<60	3216 (40.0)	729 (55.9)
≥60	4819 (60.0)	576 (44.1)
**Race**		
White	5999 (74.7)	
Black	1312 (16.3)	
Asian or Pacific Islander	674 (8.4)	
American Indian/Alaska Native	50 (0.6)	
**Sex**		
Male	4368 (54.4)	850 (65.1)
Female	3667 (45.6)	455 (34.9)
**Primary tumor site**		
Colon	7708 (95.9)	758 (58.1)
Rectum	327 (4.1)	547 (41.9)
**Grade**		
Well/Moderately	5797 (72.1)	855 (65.5)
Poorly/Undifferentiated	2238 (27.9)	294 (22.5)
Unknown		156 (12.0)
**Histology type**		
Adenocarcinoma	7427 (92.4)	1256 (96.2)
Mucinous adenocarcinoma	608 (7.6)	49 (3.8)
**AJCC T stage**		

Characteristics	SEER Database No. (%)	Chinese Registry No. (%)
T1-T2	211 (2.6)	60 (4.6)
T3-T4	7824 (97.4)	1245 (95.4)
**Radiotherapy**		
Yes	211 (2.6)	81 (6.2)
No	7824 (97.4)	1209 (92.6)
Unknown		15 (1.1)
**Chemotherapy**		
Yes	5492 (68.4)	1118 (85.7)
No	2543 (31.6)	167 (12.8)
Unknown		20 (1.5)
**Metastasectomy**		
Yes	2191 (27.3)	637 (48.8)
No	5844 (72.7)	668 (51.2)
**Diameter, cm**		
≤5	3989 (49.6)	650 (49.8)
5-10	3551 (44.2)	221 (16.9)
>10	300 (3.7)	11 (0.8)
Unknown	195 (2.4)	423 (32.4)

LNM, Lymph Nodes Metastases; SEER, Surveillance, Epidemiology, and End Results; AJCC, American Joint Committee on Cancer

2

Supplementary Table 2 Association between LNM and clinicopathological characteristics of CRLM patients in SEER database and Chinese registry.

Characteristics	SEER Database						Chinese Registry					
	Before IPTW			After IPTW			Before IPTW			After IPTW		
	Non-LNM	LNM	*P*	Non-LNM	LNM	*P*	Non-LNM	LNM	*P*	Non-LNM	LNM	*P*
**Total**	1152	6883		8101.9	8033.8		331	974		1304.8	1304.2	
**Age, years**			<0.001			0.719			0.026			0.786
<60	389(33.8)	2827(41.1)		3296.2(40.7)	3217.1(40.0)		167 (50.5)	562 (57.7)		744.0 (57.0)	731.5 (56.1)	
**Race**			0.003			0.839						
White	819(71.1)	5180(75.3)		6148.2(75.9)	6000.0(74.7)							
Black	228(19.8)	1084(15.7)		1273.3(15.7)	1310.9(16.3)							
Asian or Pacific Islander	101(8.8)	573(8.3)		641.2(7.9)	673.0(8.4)							
American Indian/Alaska Native	4(0.3)	46(0.7)		39.3(0.5)	49.9(0.6)							
**Sex**			0.093			0.362			0.145			0.675
Male	653(56.7)	3715(54.0)		4538.3(56.0)	4371.2(54.4)		227 (68.6)	623 (64.0)		826.6 (63.3)	845.2 (64.8)	
**Primary tumor site**			0.457			0.789			0.350			0.707
Colon	1100(95.5)	6608(96.0)		7757.3(95.7)	7706.9(95.9)		200 (60.4)	558 (57.3)		738.5 (56.6)	755.4 (57.9)	
**Grade**			<0.001			0.531			<0.001			0.805
Well/Moderately	989(85.9)	4808(69.9)		5749.8(71.0)	5795.1(72.1)		257 (77.6)	598 (61.4)		878.6 (67.3)	856.1 (65.6)	
Poorly/Undifferentiated	163(14.1)	2075(30.1)		2352.1(29.0)	2238.7(27.9)		53 (16.0)	241 (24.7)		292.5 (22.4)	294.2 (22.6)	

Characteristics	SEER Database						Chinese Registry					
	Before IPTW			After IPTW			Before IPTW			After IPTW		
	**Non-LNM**	**LNM**	***P***	**Non-LNM**	**LNM**	***P***	**Non-LNM**	**LNM**	***P***	**Non-LNM**	**LNM**	***P***
Unknown							21 (6.3)	135 (13.9)		133.7 (10.2)	154.0 (11.8)	
**Histology type**			0.297			0.978			0.519			0.672
Adenocarcinoma	1074(93.2)	6353(92.3)		7487.4(92.4)	7426.6(92.4)		321 (97.0)	935 (96.0)		1246.9 (95.6)	1254.8(96.2)	
**AJCC T stage**			<0.001			0.902			<0.001			0.996
T3-T4	1086(94.3)	6738(97.9)	`	7893.6(97.4)	7823.3(97.4)		298 (90.0)	947 (97.2)		1240.8(95.1)	1240.4(95.1)	
**Radiotherapy**			0.881			0.531			0.001			0.940
Yes	29(2.5)	182(2.6)		216.8(2.7)	211.2(2.6)		22 (6.6)	59 (6.1)		77.9 (6.0)	81.1 (6.2)	
No	1123(97.5)	6701(97.4)		7885.1(97.3)	7822.6(97.4)		299 (90.3)	910 (93.4)		1212.4(92.9)	1211.1(92.9)	
Unknown							10 (3.0)	5 (0.5)		14.5 (1.1)	12.1 (0.9)	
**Chemotherapy**			<0.001			0.697			0.011			0.979
Yes	734(63.7)	4758(69.1)		5589.2(69.0)	5493.0(68.4)		271 (81.9)	847 (87.0)		1122.1 (86.0)	1120.1(85.9)	
No	418(36.3)	2125(30.9)		2512.7(31.0)	2540.8(31.6)		50 (15.1)	117 (12.0)		163.2 (12.5)	166.1(12.7)	
Unknown							10 (3.0)	10 (1.0)		19.6 (1.5)	18.0 (1.4)	
**Metastasectomy**			0.304			0.598			0.450			0.788
Yes	329(28.6)	1862(27.1)		2143.9(26.5)	2190.5(27.3)		168 (50.8)	469 (48.2)		654.2 (50.1)	641.5 (49.2)	
**Diameter, cm**			<0.001			0.821			<0.001			0.997
≤5	514(44.6)	3475(50.5)		4144.6(51.2)	3991.5(49.7)		197 (59.5)	453 (46.5)		658.6 (50.5)	651.6 (50.0)	
5-10	544(47.2)	3007(43.7)		3455.2(42.6)	3546.4(44.1)		70 (21.1)	151 (15.5)		221.6 (17.0)	219.1 (16.8)	
>10	60(5.2)	240(3.5)		309.7(3.8)	301.0(3.7)		3 (0.9)	8 (0.8)		10.3 (0.8)	10.8 (0.8)	

Characteristics	SEER Database						Chinese Registry					
	Before IPTW			After IPTW			Before IPTW			After IPTW		
	Non-LNM	LNM	***P***	Non-LNM	LNM	***P***	Non-LNM	LNM	***P***	Non-LNM	LNM	***P***
Unknown	34(3.0)	161(2.3)		192.4(2.4)	195.2(2.4)		61 (18.4)	362 (37.2)		414.2 (31.7)	422.7 (32.4)	

LNM, Lymph Nodes Metastases; SEER, Surveillance, Epidemiology, and End Results; AJCC, American Joint Committee on Cancer; IPTW, Inverse Probability of Treatment Weighting.

Supplementary Table 3 Univariate and multivariate Cox analysis for CSS of CRLM patients.

Variable	SEER Database				Chinese Registry			
	Univariate analysis		Multivariate analysis		Univariate analysis		Multivariate analysis	
	HR (95% CI)	*P*	HR (95% CI)	*P*	HR (95% CI)	*P*	HR (95% CI)	*P*
**LNM**								
No	Reference		Reference		Reference		Reference	
Yes	1.54 (1.43 – 1.67)	<0.001	1.72 (1.59 – 1.86)	<0.001	1.63 (1.39 – 1.92)	<0.001	1.46 (1.24 – 1.73)	<0.001
**Age, years**								
<60	Reference		Reference		Reference			
≥60	1.52 (1.44 - 1.60)	<0.001	1.32 (1.25 – 1.39)	<0.001	1.11 (0.97- 1.26)	0.12		
**Race**								
White	Reference		Reference					
Black	1.08 (1.01 – 1.16)	0.02	1.14 (1.07 – 1.22)	<0.001				
Asian or Pacific Islander	0.84 (0.76 – 0.92)	<0.001	0.77 (0.70 - 0.85)	<0.001				
American Indian/Alaska Native	0.71 (0.50 – 1.02)	0.06	0.85 (0.60 – 1.22)	0.38				
**Sex**								
Female	Reference		Reference		Reference			
Male	0.92 (0.88 – 0.97)	0.002	0.93 (0.88 – 0.98)	0.004	0.94 (0.85 – 1.12)	0.70		
**Primary tumor site**								
Colon	Reference		Reference		Reference			

Variable	SEER Database				Chinese Registry			
	Univariate analysis		Multivariate analysis		Univariate analysis		Multivariate analysis	
	HR (95% CI)	*P*	HR (95% CI)	*P*	HR (95% CI)	*P*	HR (95% CI)	*P*
Rectum	0.81 (0.71 - 0.92)	0.002	0.91 (0.80 – 1.04)	0.18	0.98 (0.86 – 1.11)	0.74		
**Grade**								
Poorly/Undifferentiated	Reference		Reference		Reference		Reference	
Well/Moderately	0.62 (0.59 – 0.66)	<0.001	0.64 (0.61 – 0.68)	<0.001	0.70 (0.60 – 0.82)	<0.001	0.75 (0.64 - 0.88)	<0.001
Unknown					1.44 (1.16 - 1.78)	<0.001	0.88 (0.69 – 1.12)	0.30
**Histology type**								
Adenocarcinoma	Reference		Reference		Reference			
Mucinous adenocarcinoma	1.35 (1.23 – 1.48)	<0.001	1.24 (1.13 – 1.36)	<0.001	1.68 (1.20 – 2.35)	0.002		
**AJCC T stage**								
T1-T2	Reference		Reference		Reference		Reference	
T3-T4	1.48 (1.24 - 1.76)	<0.001	1.45 (1.21 – 1.73)	<0.001	2.66 (1.78 – 4.00)	<0.001	1.92 (1.27 – 2.91)	0.002
**Radiotherapy**								
No	Reference		Reference		Reference		Reference	
Yes	0.72 (0.61 – 0.85)	<0.001	0.88 (0.75 – 1.04)	0.14	0.92 (0.71 – 1.20)	0.54	0.88 (0.68 – 1.15)	0.36
Unknown					1.79 (1.01 – 3.17)	<0.050	1.92 (1.02 – 3.59)	0.04
**Chemotherapy**								
No	Reference		Reference		Reference		Reference	
Yes	0.36 (0.34 – 0.37)	<0.001	0.36 (0.34 – 0.38)	<0.001	0.71 (0.59 – 0.87)	<0.001	0.95 (0.77 – 1.18)	0.65

Variable	SEER Database				Chinese Registry			
	Univariate analysis		Multivariate analysis		Univariate analysis		Multivariate analysis	
	**HR (95% CI)**	***P***	**HR (95% CI)**	***P***	**HR (95% CI)**	***P***	**HR (95% CI)**	***P***
Unknown					1.24 (0.71 – 2.16)	0.44	1.14 (0.62 – 2.10)	0.68
**Metastasectomy**								
No	Reference		Reference		Reference		Reference	
Yes	0.69 (0.65 – 0.73)	<0.001	0.75 (0.71 – 0.80)	<0.001	0.36 (0.31 – 0.41)	<0.001	0.42 (0.37 – 0.49)	<0.001
**Diameter, cm**								
≤5	Reference		Reference		Reference		Reference	
5-10	1.12 (1.06- 1.18)	<0.001	1.11 (1.06 – 1.17)	<0.001	1.36 (1.12 – 1.64)	0.002	1.17 (0.96 – 1.42)	0.11
>10	1.28 (1.11 – 1.46)	<0.001	1.31 (1.14 – 1.50)	<0.001	3.64 (1.94 – 6.82)	<0.001	2.23 (1.17 – 4.27)	0.02
Unknown	1.00 (0.85 – 1.19)	0.98	1.00 (0.84 – 1.18)	0.97	2.20 (1.90 – 2.54)	<0.001	1.54 (1.30 – 1.83)	<0.001

LNM, Lymph Nodes Metastases; SEER, Surveillance, Epidemiology, and End Results; AJCC, American Joint Committee on Cancer; HR, Hazard Ratio; CI, Confidence Interval

8
